# Multi-isolate GWAS identifies broad-spectrum and isolate-specific resistance-associated loci for Septoria tritici blotch in synthetic hexaploid wheat

**DOI:** 10.1007/s00122-026-05423-2

**Published:** 2026-10-20

**Authors:** Anisa Blower, Richard Horsnell, Phil M. Howell, Tally I. C. Wright, Rumiana Ray, Stephen Rawsthorne, Fiona J. Leigh, Kostya Kanyuka

**Affiliations:** 1 University of Nottingham, Plant Sciences Building, Sutton Bonnington Campus, Nottingham, LE12 5RD UK; 2 Park Farm Campus, Villa Road, Niab, Histon, Cambridge, CB24 9AT UK; 3 Morley Business Centre, The Morley Agricultural Foundation, Deopham Road, Morley St Botolph, Wymondham, NR18 9DF UK

## Abstract

***Key message*:**

**Synthetic hexaploid wheat harbours STB resistance-associated loci with multi-isolate and isolate-specific effects, with partly independent genetic control of pycnidia suppression and necrosis.**

**Abstract:**

Septoria tritici blotch (STB), caused by *Zymoseptoria tritici*, is a major constraint on wheat production. Erosion of host resistance and declining fungicide efficacy highlight the need to exploit new resistance sources, including synthetic hexaploid wheat (SHW). We used a three-family nested association mapping (NAM) population derived from Niab SHW donors (SHW.035, SHW.054, and SHW.075) crossed with the UK variety Robigus to dissect the genetic basis of STB resistance under controlled conditions. Seedlings were challenged with five UK *Z. tritici* isolates differing in virulence, and disease progress was quantified separately as necrosis (AUDPC_N) and pycnidia coverage (AUDPC_P). Many SHW-derived lines showed strong suppression of pycnidia despite visible necrosis, consistent with partial genetic decoupling of pathogen reproduction and host tissue damage. GWAS identified a major multi-isolate association on chromosome 3D (*qSTB-3D.1*), contributed by SHW.035 and detected in response to four of the five isolates, defining a 23.7 Mb candidate resistance-associated interval. This interval encompasses the *Stb16q* region but also contains other plausible resistance candidates, including a WAK close to the strongest association at 609.21 Mb; the causal basis therefore remains unresolved. Additional isolate-specific resistance-associated intervals were detected on chromosomes 1B, 2D, 3A, and 6D. In a BC_2_F_2_ population derived from a resistant SHW.035-derived NAM line, pycnidia suppression segregated in a 3:1 ratio consistent with a single dominant factor, whereas necrosis showed more complex inheritance. These findings show that Niab SHWs are a valuable source of STB resistance and highlight the potential to select for reduced pathogen reproduction independently of visible leaf damage.

**Supplementary Information:**

The online version contains supplementary material available at https://doi.org/10.1007/s00122-026-05423-2.

## Introduction

*Zymoseptoria tritici*, previously known as *Septoria tritici* and *Mycosphaerella graminicola*, is a filamentous ascomycete fungus and the causal agent of Septoria tritici blotch (STB) in wheat. The disease is particularly prevalent in temperate regions with cool, humid climates, including Northern Europe and North America, where severe epidemics can reduce grain yield and quality by up to 50% in susceptible varieties under favourable conditions (Fones and Gurr 2015; Castro and Simón 2017). Disease management is further complicated by the large and genetically diverse populations of *Z. tritici*, which promote the rapid evolution of fungicide resistance and the breakdown of host resistance genes (McDonald et al. 2022).

In Europe, STB management accounts for approximately 70% of the total fungicide market in wheat, at an estimated annual cost of €1.0 billion (Torriani et al. 2015). However, the effectiveness of fungicide-based control is declining because of the widespread emergence of fungicide resistance, including reduced sensitivity to azoles (Heick et al. 2020; Jørgensen et al. 2021), strobilurins (Torriani et al. 2009), and succinate dehydrogenase inhibitors (Dooley et al. 2016; Hagerty et al. 2017, 2021). Consequently, STB remains a major challenge to sustainable wheat production. The development of new wheat varieties with durable genetic resistance, combined with integrated disease management, is therefore widely regarded as an effective long-term strategy for STB control.

Twenty-three major STB resistance (*Stb*) genes have been identified in wheat (Brown et al. 2015; Yang et al. 2018; Langlands-Perry et al. 2021), in addition to more than 160 resistance-associated quantitative trait loci (QTL) (Brown et al. 2015). Several qualitative resistance genes have been cloned, including *Stb6*, encoding a wall-associated kinase-like protein (WAK) (Saintenac et al. 2018), *Stb16q*, encoding a cysteine-rich receptor-like kinase (CRK) (Saintenac et al. 2021), and *Stb15*, encoding a lectin receptor-like kinase (LecRK) (Hafeez et al. 2025). While many *Stb* genes confer isolate-specific resistance, some, including *Stb10*, *Stb11*, *Stb12*, *Stb16q*, *Stb17*, and *Stb19*, provide resistance to diverse field isolates, with synthetic hexaploid wheat (SHW) emerging as a particularly promising source of such resistance (Tidd et al. 2023).

SHW, generated by hybridising tetraploid durum wheat with the wild D-genome progenitor *Aegilops tauschii* (Wright et al. 2024), has enabled the transfer of drought tolerance (Mokhtari et al. 2022) and disease resistance (Ogbonnaya et al. 2008; Jighly et al. 2016; Blower et al. 2025) from wild relatives into bread wheat. Several STB resistance loci, including *Stb5* (Arraiano et al. 2001), *Stb8* (Adhikari et al. 2003), *Stb16q* (Saintenac et al. 2021), and *Stb17* (Tabib Ghaffary et al. 2012), were identified in SHW backgrounds, underlining the value of SHW as a reservoir of useful alleles for STB resistance improvement.

To dissect complex resistance traits derived from exotic germplasm, structured multi-parent populations such as nested association mapping (NAM) populations combine multiple donor genomes within a common elite background, enabling the detection of both major and minor loci, while reducing confounding effects of population structure (Yu et al. 2008). Using an SHW-derived NAM population developed from crosses with the elite UK variety Robigus, Wright et al. (2024) identified novel SHW-derived variation for flowering time, plant height, and yellow rust resistance.

In the present study, a subset of this SHW-derived NAM population (Wright et al. 2024) was used to investigate the genetic basis of STB resistance by combining artificial seedling inoculation assays with genome-wide association analysis. The *Z. tritici* isolate panel was selected on the basis of prior virulence profiling against differential wheat lines carrying major *Stb* genes and their combinations and was collectively virulent on 19 of 23 characterised resistance genes (Tidd et al. 2023), enabling evaluation of resistance breadth against diverse pathogen genotypes. Although resistance expressed at the adult plant stage can involve additional loci and environmental interactions (Thauvin et al. 2024), controlled seedling assays provide a reproducible framework for detecting isolate-specific resistance associations under defined conditions. These findings provide a foundation for the targeted deployment of SHW-derived diversity to improve STB resistance.

## Materials and methods

### Plant material and population development

A three-family NAM population was used to investigate genetic resistance to *Z. tritici* in hexaploid wheat. The population was developed from crosses between the elite UK winter wheat variety Robigus and three primary SHW lines: SHW.035, SHW.054, and SHW.075. Population development followed a backcrossing and single-seed descent strategy, as described in detail by Wright et al. (2024). The final population consisted of three nested families (Table 1) comprising 201 backcross-derived lines. All 201 NAM lines were genotyped at the BC_1_F_5_ generation, together with the four parents: the recurrent parent Robigus and the three SHW donor parents. Phenotypic evaluations were conducted at the BC_1_F_7_ generation on 180 NAM lines, together with five reference lines: Robigus, the three SHW donor parents, and the highly susceptible UK winter wheat variety KWS Cashel. Genetic analyses were conducted on 171 NAM lines, together with the four parents, for which genotype and phenotype data of sufficient quality were available.

**Table 1:** Details of the three-family NAM population used in this study, including population size and parental origin

Population name	Total lines genotyped & phenotyped (*n*)	Synthetic parent	SHW DD donor	SHW AABB donor
NAM.144	61	SHW.035	WX224	Biensur
NAM.212	48	SHW.054	Ent.392	Biensur
NAM.233	62	SHW.075	Ent.404	Hoh-501

### Fungal material

Five *Z. tritici* isolates were selected from a panel of 93 previously characterised UK field isolates (Tidd et al. 2023). These isolates were originally cultured from STB lesions collected from hexaploid wheat grown in the UK between 2015 and 2017. The isolates used in this study were HT-18, HT-44, HT-53, HT-74, and HT-96. All isolates were maintained as pure blastospore stocks in 50% (v/v) glycerol and stored at − 80 °C.

### Inoculum preparation and inoculation

Susceptibility of wheat lines was assessed using a method adapted from Tidd et al. (2023). *Z. tritici* isolates were streaked from glycerol stocks onto antibiotic-free yeast peptone dextrose (YPD) agar (Formedium) and incubated in the dark for 5–7 days at 15 °C before inoculation. Fungal blastospores were harvested from plates using sterile loops and transferred to tubes containing 5 mL sterile distilled water. The spore suspension was filtered through autoclaved Miracloth (Merck). Spore concentration was determined using a haemocytometer from the mean of four replicated counts, and suspensions were adjusted to 5 × 10^6^ spores per mL before inoculation. Tween 20 was added to a final concentration of 0.05% (v/v) to improve leaf wetting and reduce spore aggregation.

Wheat seedlings were grown for approximately 3 weeks under controlled conditions before inoculation. The second fully expanded seedling leaf was fixed to an aluminium platform with the adaxial surface facing upwards and secured using rubber bands and double-sided sticky tape. Cotton buds soaked in the spore suspension were used to distribute inoculum evenly across the leaf surface. A sterile solution of 0.05% (v/v) Tween 20 in distilled water was used as a mock-inoculation control. KWS Cashel, a wheat variety lacking established *Stb* resistance genes, was used as a susceptible control.

After inoculation, each half-tray containing inoculated plants was placed in a high-humidity box for three days. For each wheat genotype × *Z. tritici* isolate combination, represented within an experiment, approximately 5–7 individual plants were inoculated. The main NAM screen comprised 11 experimental batches, each containing different subsets of 8–79 genotypes and multiple isolates; individual isolates were represented in 8–10 batches, and some genotype × isolate combinations were repeated across batches. KWS Cashel and Robigus were included throughout as reference genotypes, together with the relevant SHW parent or parents. Five additional parent-only batches provided further observations of the SHW parental responses across the isolate panel. The resulting dataset was therefore unbalanced with respect to genotype, isolate and batch representation. Plants were maintained for 29 days post inoculation (dpi) and screened without formal randomisation.

### Phenotypic analysis

#### Visual phenotypic trait evaluation

**Fig. 1:**
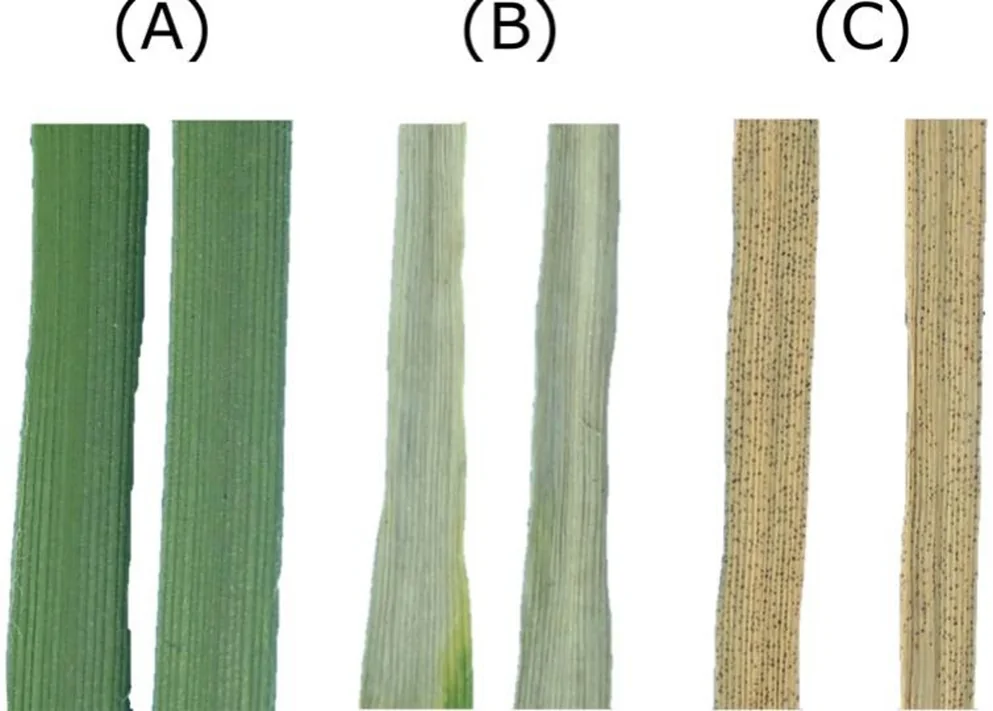
Seedling phenotypes following inoculation with *Zymoseptoria tritici*. (**A**) Resistant phenotype showing no necrosis and absence of pycnidia. (**B**) Intermediate phenotype with visible necrosis but no pycnidia development. (**C**) Susceptible phenotype showing extensive necrosis and abundant pycnidia formation

Disease symptom development on inoculated leaves was scored visually at 2–3-day intervals from 10- to 29-dpi. Percentage leaf coverage by necrosis, chlorosis, and pycnidia was scored in 20-percentage-point increments from 0 to 100%. Representative examples illustrating necrosis and pycnidia development are shown in Fig. 1.

The area under the disease progress curve (AUDPC) was calculated for each plant–pathogen interaction according to Simko and Piepho (2012):

$$\:AUDPC=\:\frac{\sum\:({t}_{i+1}-{t}_{i})({y}_{i}+{y}_{i+1})}{2}$$

where *y*_*i*_ and *y*_*i+1*_ are the percentages of leaf area affected at consecutive observations *i* and *i + 1* and ($\:{t}_{i+1}-{t}_{i}$) is the number of days between observations. AUDPC values were calculated separately for necrosis (AUDPC_N), and pycnidia (AUDPC_P). The same scoring schedule was applied across all experimental batches.

#### Statistical analysis of AUDPC data

AUDPC_N and AUDPC_P were analysed using linear mixed models in R v.4.3.2 (R Core Team 2021), using lme4 (Bates et al. 2015), lmerTest (Kuznetsova et al. 2017) and emmeans (Lenth 2023). Analyses were conducted using untransformed AUDPC values. Model diagnostics were examined, and square-root, cube-root and log1*p* transformations were evaluated as sensitivity analyses. As these transformations did not materially alter the principal conclusions, results are presented on the untransformed AUDPC scale.

To test for differences among the three NAM families and five *Z. tritici* isolates, NAM family, isolate and their interaction were fitted as fixed effects. Genotype and genotype × isolate were fitted as random effects. Because different subsets of genotypes and isolates were represented across experimental batches, and some genotype × isolate combinations were repeated across batches, batch, genotype × batch, isolate × batch and genotype × isolate × batch were also fitted as random effects to account for the unbalanced experimental structure:

$$\begin{aligned}\:{y}_{ijkl}&=\mu\:+{f}_{l}+{s}_{k}+(f\times\:s{)}_{lk}+{G}_{i}+(G\times\:S{)}_{ik}+{B}_{j}+(G\times\:B{)}_{ij}\\&\quad+(B\times\:S{)}_{jk}+(B\times\:G\times\:S{)}_{jik}+{e}_{ijkl}\end{aligned}$$

where $\:{y}_{ijkl}$ is the AUDPC value of the *i*-th genotype in the *j*-th batch, inoculated with the *k*-th isolate and belonging to the *l*-th NAM family; $\:\mu\:\:$is the overall mean; $\:{f}_{l}\:$is the fixed effect of NAM family; $\:{s}_{k}$ is the fixed effect of isolate; $\:(f\times\:s{)}_{lk}\:$is the fixed family × isolate interaction; $\:{G}_{i}$ is the random effect of genotype; $\:(G\times\:S{)}_{ik}$is the random genotype × isolate interaction; $\:{B}_{j}$ is the random effect of batch; $\:(G\times\:B{)}_{ij}$ is the random genotype × batch interaction; $\:(B\times\:S{)}_{jk}$ is the random batch × isolate interaction; $\:(B\times\:G\times\:S{)}_{jik}$ is the random batch × genotype × isolate interaction; and $\:{e}_{ijkl}$ is the residual error term.

Models were fitted using restricted maximum likelihood (REML), and significance of fixed effects was assessed using Satterthwaite approximation of denominator degrees of freedom. Pairwise comparisons of estimated marginal means (EMMs) were performed using emmeans, with *p*-values adjusted using the Benjamini–Hochberg procedure. Adjusted *p* < 0.05 was considered significant.

To summarise genotype responses across the five isolates, a separate model was fitted with genotype and isolate as fixed effects and genotype × isolate, batch, genotype × batch, batch × isolate and batch × isolate × genotype as random effects:

$$\begin{aligned}\:{y}_{ijk}&=\mu\:+{G}_{i}+{s}_{k}+(G\times\:S{)}_{ik}+{B}_{j}+(G\times\:B{)}_{ij}+(B\times\:S{)}_{jk}\\&\quad+(B\times\:G\times\:S{)}_{jik}+{e}_{ijk}\end{aligned}$$

The notation follows that defined above, with $\:{G}_{i}\:$ representing the fixed effect of Genotype. Adjusted genotype means were obtained using emmeans, with equal weighting across the five isolates. KWS Cashel, Robigus and the three SHW founders were included to obtain adjusted reference estimates for graphical presentation but were excluded from formal comparisons among the three NAM families.

#### Estimation of genotypic effects

For isolate-specific analyses, best linear unbiased estimates (BLUEs) for AUDPC_N and AUDPC_P were estimated separately for each *Z. tritici* isolate (HT-18, HT-44, HT-53, HT-74, and HT-96). Genotype was fitted as a fixed effect and batch and genotype × batch as random effects using the following mixed-effects model:

$$\:{y}_{ij}=\mu\:+{g}_{i}+{B}_{j}+(g\times\:B{)}_{ij}+{e}_{ij}$$

The notation follows that defined above. Models were fitted using REML, and BLUEs were extracted using emmeans. For each isolate, BLUEs were calculated from untransformed AUDPC values and used in downstream analyses. For graphical comparison with the NAM material, KWS Cashel and Robigus were represented by their isolate-specific genotype BLUEs. For each isolate, the SHW reference value was calculated as an equal-weight linear contrast of the adjusted genotype estimates for SHW.035, SHW.054 and SHW.075. The corresponding 95% confidence interval was derived from the estimated variance of this model-based contrast. Relationships among isolate-specific BLUEs were evaluated using Spearman’s rank correlation coefficients. Correlations were calculated using Hmisc (Harrell et al. 2025) and visualised using corrplot (Wei and Simko 2024). *P*-values for the 45 pairwise correlations among the ten isolate-specific AUDPC traits were adjusted using the Benjamini–Hochberg procedure, with adjusted *p* < 0.05 considered significant.

#### Broad-sense heritability estimation

Broad-sense heritability ($\:{H}^{2}$) was estimated separately for each isolate and trait using the same isolate-specific model structure but with genotype fitted as a random effect to obtain best linear unbiased predictors (BLUPs) and variance components. Heritability was calculated using the generalised approach of Cullis et al. (2006):

$$\:{H}_{\mathrm{Cullis}}^{2}=1-\frac{\overline{\mathrm{PEV}}}{2{\sigma}_{G}^{2}}$$

where $\:\overline{\mathrm{PEV}}\:$ is the mean prediction error variance of pairwise differences between genotype BLUPs, and $\:{\sigma}_{G}^{2}\:$ is the estimated genotypic variance. This approach accounts for unequal replication and missing data in unbalanced experimental design. Heritability analyses were conducted in R using sommer (Covarrubias-Pazaran 2016).

### Genotypic analysis

#### DNA extraction and genotyping

Genomic DNA was previously extracted from seedling leaf tissue of the NAM population and parental lines using a modified CTAB protocol (Fulton et al. 1995) as described in Wright et al. (2024). Genotyping was conducted at the University of Bristol using the Axiom 35K Wheat Breeders’ SNP array (Thermo Fisher Scientific), which assays 35,143 SNP markers (Allen et al. 2017). All 201 NAM lines were genotyped at the BC_1_F_5_ generation, together with their recurrent parent Robigus and the three SHW donor parents, SHW.035, SHW.054, and SHW.075.

#### Initial genotype calling and marker filtering

Genotype calling and initial quality control were performed in Axiom Analysis Suite v.5.4 (Thermo Fisher Scientific) following procedures adapted from Wright et al. (2024). An inbred penalty of 4 was applied to account for the highly inbred nature of the material. Samples failing standard quality thresholds (DishQC ≥ 0.80, call rate ≥ 95%) were excluded. Markers were filtered on the basis of Axiom cluster classification and genotype class representation. Monomorphic markers, markers with poor call rates, and off-target variants were excluded. Segregating markers were retained only when clear genotype clustering was observed, and the minor genotype class was represented by at least eight individuals. All retained markers were visually inspected to confirm clustering quality, and markers showing ambiguous clustering were discarded. Following these filtering steps, the dataset comprised 8,868 SNP markers across 202 genotypes that passed the quality control thresholds. These 202 genotypes included NAM lines with genotype files available, Robigus, and the three SHW donor parents, and were counted before the removal of additional genotypes during downstream quality control steps outside Axiom Analysis Suite.

#### Downstream quality control and imputation

Further genotype quality control was conducted in R v.4.3.2 (R Core Team 2021). SNP markers were removed if they exhibited > 7% missing data, > 7% heterozygosity, or fewer than 12 individuals homozygous for the minor allele (**Supplementary Fig. S1**). Genotypes exceeding the same thresholds for missing data or heterozygosity were also excluded.

Principal coordinate analysis (PCoA) was used to identify potential outlier genotypes, both across the full NAM population and within each nested family (**Supplementary Fig. S2**). Pearson correlation coefficients among genotype pairs were used to detect highly similar or potentially duplicated samples, including selfed individuals or lines excessively similar to parental controls. Erroneous genotypes were identified and removed.

For the remaining lines, missing genotype data were imputed using the random forest algorithm implemented in missForest (Stekhoven and Bühlmann 2012), using 200 trees per forest. After imputation, the final dataset comprised 8,040 SNP markers across 187 NAM genotypes, together with four parental controls.

#### Physical marker positioning and LD-based reordering

Physical positions for 5,327 SNP markers were obtained from the IWGSC RefSeq v.1.0 wheat genome assembly (IWGSC 2018) using coordinates from the dataset described by Wright et al. (2024). For the remaining 2,713 unmapped markers, SNP probe sequences from the Axiom 35 K Wheat Breeders’ array were retrieved from CerealsDB (Wilkinson et al. 2016) and aligned to the reference genome using BLAST+ (Camacho et al. 2009). For markers with a single high-confidence BLAST hit, physical positions were assigned as the midpoint between alignment coordinates, enabling placement of 496 additional markers. Remaining unmapped markers were anchored using linkage disequilibrium (LD) with mapped markers, assigning positions when strong LD (*R*² > 0.7) and consistent BLAST hits supported a chromosomal location. Marker placement was further refined using LD-based binning (*R*² > 0.5) to improve collinearity. Markers lacking reliable placement were excluded from downstream analyses. Marker ordering and map consistency were evaluated using LD heatmaps generated using LDheatmap (Shin et al. 2006) (**Supplementary Figs. S3 and S4**). After marker positioning and filtering, 7,032 SNP markers were retained for analysis.

#### Population structure analysis

Population structure within the NAM population, including the recurrent parent Robigus and the SHW founders, was assessed using genome-wide SNP data. To reduce marker redundancy, markers were thinned by removing one marker from each pair with strong correlation (|*r*| ≥ 0.9). Population structure was visualised using principal component analysis (PCA) based on a genetic distance matrix calculated from the filtered marker set. Analyses were conducted in R and plots were generated using ggplot2 (Wickham 2016). Principal components and pedigree information were used to define covariates included in the genome-wide association study.

#### Genome-wide association study

Genome-wide association study (GWAS) was performed on 171 phenotyped NAM lines, for which high-quality genotype data were available, together with the four parental controls (**Supplementary Table ****S1**). GWAS was performed using an additive Q + K mixed model implemented in GWASpoly (Rosyara et al. 2016). Population structure (Q) was accounted for using fixed-effect covariates corresponding to family and tetraploid donor, together with the first ten principal components (**Supplementary Fig. S5**), while genetic relatedness (K) was controlled using a marker-derived kinship matrix estimated with the GWASpoly function *set.K*. Leave-one-chromosome-out (LOCO) correction was not applied. Prior to GWAS, markers were skimmed to remove non-unique markers (|*r*| = 1) to reduce redundancy and avoid inflation of kinship estimates (**Supplementary Table ****S2**). Model suitability was assessed by inspection of quantile–quantile (QQ) plots and estimation of genomic inflation factors (*λ*; Devlin and Roeder 1999), and the final model settings were applied consistently across all traits. Significance thresholds were determined using the GWASpoly *set.threshold* function, applying both false discovery rate correction (FDR; *q* = 0.05) (Benjamini and Hochberg 1995) and permutation-derived thresholds based on 1,000 permutations (*α* = 0.05) (Churchill and Doerge 1994). The permutation-derived threshold was used as the primary significance criterion, while associations meeting the FDR threshold but not the permutation threshold were retained as additional FDR-supported associations. Candidate QTLs were defined based on −log10(*p*) values, allele effect direction (using Robigus as the reference allele), and physical marker position. LD between markers was calculated as *r²* using the GWASpoly *LD.plot* function. LD decay was assessed by plotting *r²* against physical distance, and the LD decay threshold was defined as the physical distance at which *r²* declined to 0.2 (**Supplementary Fig. S6**). Candidate QTL regions were defined around significant association peaks using the LD structure and flanking significant SNP markers. QTLs retained for each phenotype were fitted jointly in a multiple-QTL model using GWASpoly fit.QTL function to estimate the proportion of phenotypic variation attributable to each locus.

#### Identification of candidate genes

Candidate genes underlying significant resistance-associated intervals were identified by analysing gene content within QTL intervals defined by the outermost significant flanking SNPs and extended to include one additional marker on each side. Physical coordinates were based on the IWGSC RefSeq v.1.1 wheat genome assembly. Gene models were retrieved from the Ensembl Plants BioMart database using biomaRt (Durinck 2009), extracting gene identifiers, genomic coordinates, functional descriptions, and InterPro domain annotations. Candidate genes encoding protein classes previously associated with plant disease resistance, such as nucleotide-binding leucine-rich repeat proteins (NLRs), wall-associated kinase-like proteins (WAKs), cysteine-rich receptor-like kinases (CRKs), lectin receptor-like kinases (LecRKs), leucine-rich repeat receptor-like kinases (LRR-RLKs), receptor-like proteins (RLPs), and other kinase-containing proteins, were prioritised.

### Development and screening of the BC_2_F_2_ mapping population

To investigate the inheritance of resistance identified in the NAM.144 family, genotype SHW.BC.144.12.1.4.1 (BC₁F₇; SHW.035 × Robigus) was selected as the resistant parent on the basis of its consistent resistance to multiple *Z. tritici* isolates. SHW.BC.144.12.1.4.1 was crossed with the susceptible parent Robigus (female) under glasshouse conditions, and the resulting F₁ progeny were selfed to produce a BC_2_F₂ population.

A total of 256 BC_2_F_2_ seedlings, derived from a single crossing event, were evaluated for resistance alongside the parental controls SHW.BC.144.12.1.4.1 and Robigus. The population was inoculated with *Z. tritici* isolate HT-74 and disease phenotypes were assessed at 29-dpi. Phenotyping was conducted in a single experimental batch, with twelve parental control plants included. Because pycnidia coverage was scored in 20-percentage-point increments, individuals with 0% pycnidia coverage were classified as resistant and those with ≥ 20% coverage as susceptible. For necrosis, individuals with 0–20% leaf coverage were classified as resistant and those with ≥ 40% coverage as susceptible. Segregation of pycnidia resistance was tested against a 3:1 Mendelian expectation using a chi-squared (*χ*²) goodness-of-fit test. Necrosis segregation was compared with simple 3:1 and 1:3 expectations.

## Results

### Phenotypic analysis of resistance to *Z. tritici*

Resistance to STB was assessed in the three-family NAM population under controlled growth-room conditions following separate inoculations with five *Z. tritici* isolates (HT-18, HT-44, HT-53, HT-74, and HT-96). Disease progression was assessed at several timepoints between 10- and 29-dpi by scoring the percentage leaf area covered by pycnidia and necrosis. These scores were used to calculate AUDPC_P and AUDPC_N.

#### Isolate-specific differences in disease development across host backgrounds

**Fig. 2:**
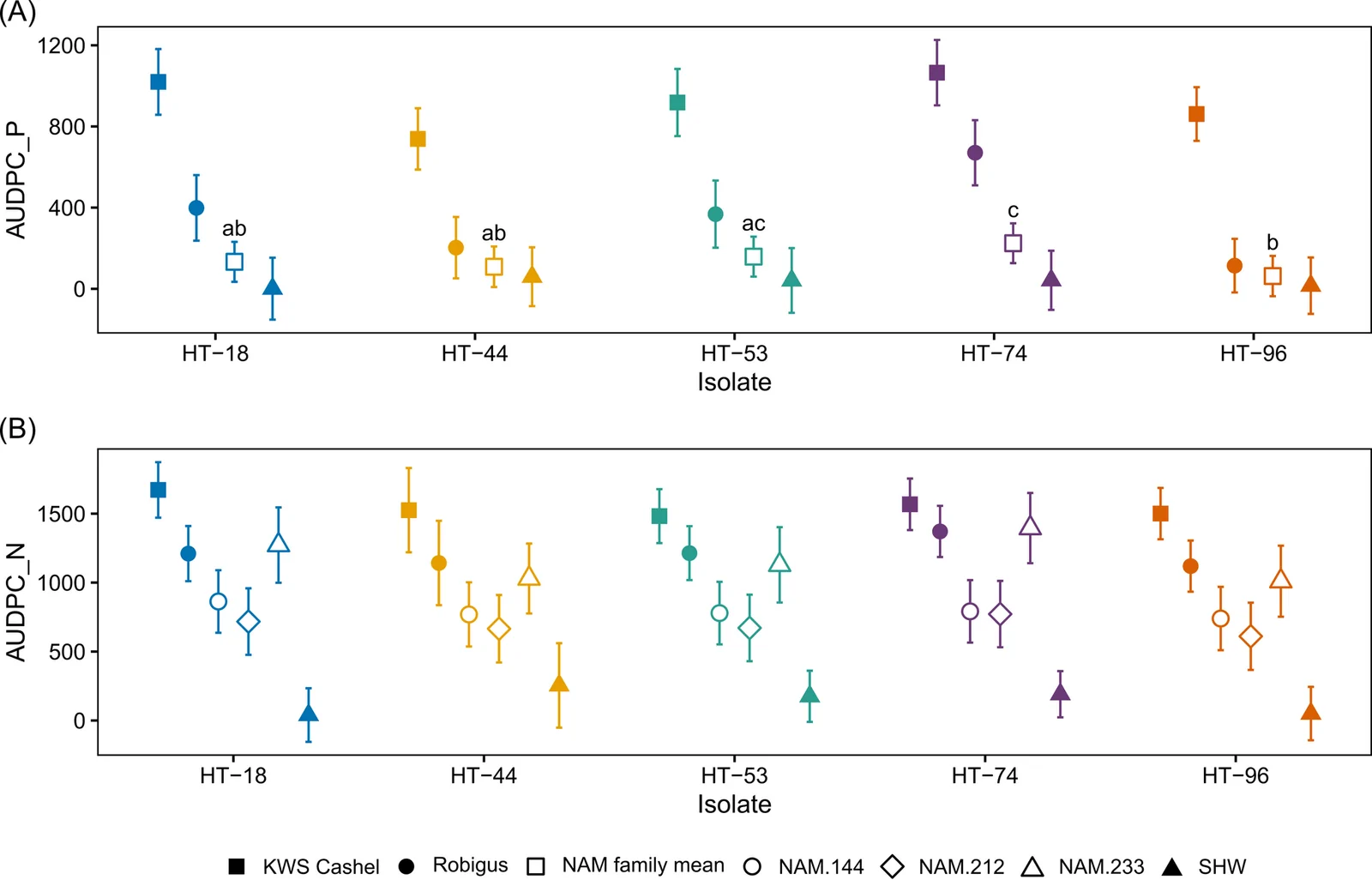
Isolate-specific differences in STB development across wheat host backgrounds. Area under the disease progress curve for pycnidia development (AUDPC_P; **A**) and necrosis (AUDPC_N; **B**) following inoculation with five *Zymoseptoria tritici* isolates (HT-18, HT-44, HT-53, HT-74, and HT-96). KWS Cashel and Robigus are shown as adjusted reference-genotype estimates. The SHW estimate represents an equal-weight contrast of the adjusted genotype estimates for SHW.035, SHW.054, and SHW.075 and is shown as a descriptive reference; its 95% confidence interval is derived from the estimated variance of this model-based contrast. The SHW estimate represents the equal-weight response of SHW.035, SHW.054, and SHW.075 and is shown as a descriptive reference. For AUDPC_P, the NAM estimate represents the estimated marginal isolate response averaged equally across NAM.144, NAM.212, and NAM.233. For AUDPC_N, the three NAM families are shown separately because a significant family × isolate interaction was detected. Different letters indicate significant Benjamini–Hochberg-adjusted pairwise comparisons where shown (adjusted *p* < 0.05). Error bars indicate 95% confidence intervals for model-based estimates

Clear isolate-specific differences in disease development were observed across host genetic backgrounds (Fig. 2). Successful inoculation was confirmed for all five isolates, each of which produced substantial disease on the susceptible control KWS Cashel. In Robigus, the extent of pycnidia and necrosis development also varied among isolates with HT-74 generally producing the greatest pycnidia development and HT-96 the lowest.

Across the NAM population, isolate had a significant effect on AUDPC_P (*P* = 0.00130), whereas NAM family (*P* = 0.812) and the family × isolate interaction (*P* = 0.553) were not significant. Isolate responses for AUDPC_P were therefore summarised across the three NAM families (Fig. 2). Mean AUDPC_P followed the numerical order HT-74 > HT-53 > HT-18 > HT-44 > HT-96. For AUDPC_N, NAM family had a significant effect (*P* = 3.03 × 10⁻⁷) and a significant family × isolate interaction was detected (*P* = 0.00376), whereas the overall isolate effect was not significant (*P* = 0.0788). The three NAM families are therefore shown separately in Fig. 2. The significant interaction indicated that the magnitude of differences among NAM families varied according to isolate.

#### NAM family-level variation in pycnidia and necrosis severity

**Fig. 3:**
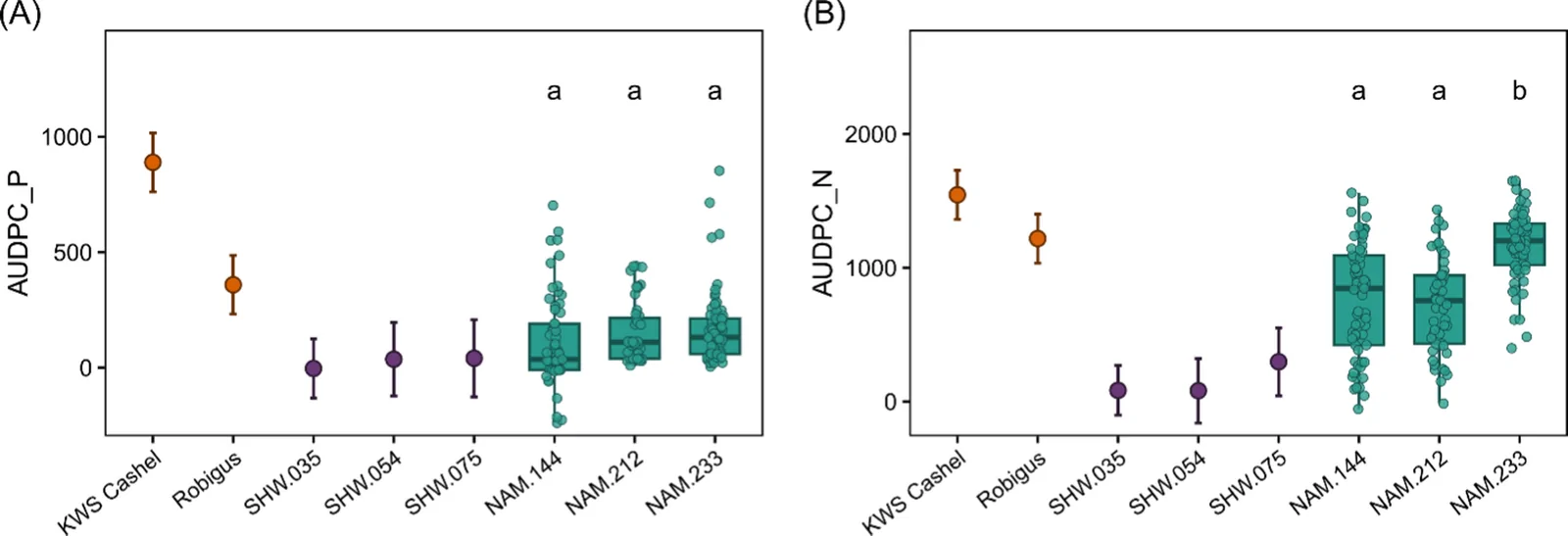
Adjusted genotype responses across five *Zymoseptoria tritici* isolates. Adjusted genotype means for pycnidia development (AUDPC_P; **A**) and necrosis (AUDPC_N; **B**), estimated with equal weighting across HT-18, HT-44, HT-53, HT-74, and HT-96. Thus, each genotype value represents its adjusted mean response across the complete five-isolate panel. KWS Cashel, Robigus, and the three SHW founders are shown as reference-genotype estimates. Points within NAM.144, NAM.212, and NAM.233 represent adjusted genotype means, with boxplots showing their distributions. Reference genotypes were not included in formal NAM-family comparisons. Different letters indicate significant differences among NAM families based on Benjamini–Hochberg-adjusted pairwise comparisons (adjusted *p* < 0.05)

To evaluate overall variation in STB resistance, adjusted genotype responses for AUDPC_P and AUDPC_N were estimated across the five-isolate panel, with equal weighting of isolates (Fig. 3). KWS Cashel, Robigus, and the three SHW founders (SHW.035, SHW.054, and SHW.075) are shown as reference genotypes, whereas formal statistical comparisons were restricted to SHW-derived NAM families (NAM.144, NAM.212, and NAM.233). KWS Cashel and Robigus showed high overall AUDPC_P, whereas the three SHW founders strongly restricted pycnidia development. Despite strong restriction of pycnidiation, the SHW founders developed varying amounts of necrosis, particularly SHW.075.

When adjusted genotype responses were averaged across the five isolates, the three NAM families did not differ significantly for AUDPC_P, although considerable variation was observed among individual genotypes within each family. In contrast, overall AUDPC_N differed among NAM families, with NAM.144 and NAM.212 showing lower necrosis than NAM.233. The significant family × isolate interaction nevertheless indicated that the magnitude of these family differences varied among isolates. Several individual SHW-derived NAM lines showed consistently low necrosis and near-complete suppression of pycnidia across all five isolates. These lines therefore represent useful candidates for subsequent genetic analysis.

#### Heritability of isolate-specific disease responses

Broad-sense heritability of disease responses was estimated separately for each *Z. tritici* isolate and disease component using the generalised heritability approach of Cullis et al. (2006), based on genotype BLUPs obtained from mixed-model analysis (Table 2). Heritability for AUDPC_N was consistently moderate to high across isolates, ranging from 0.76 to 0.88. Heritability for AUDPC_P ranged from 0.65 to 0.79. For AUDPC_P, the highest heritability was observed for isolate HT-74 (*H*² = 0.79), whereas the lowest was observed for HT-96 (*H*² = 0.65). Across all isolates, heritability estimates were consistently higher for AUDPC_N than for AUDPC_P. Corresponding estimates of genotypic variance and mean prediction error variance are presented in Table 2.

**Table 2:** Broad-sense heritability (*H*²_*cullis*_) estimates for necrosis (AUDPC_N) and pycnidia (AUDPC_P)

Isolate	Trait	H^2^_cullis_	V_g_	PEV
HT-18	AUDPC_N	0.83	186910.20	64200.08
HT-44	AUDPC_N	0.76	203191.90	98541.13
HT-53	AUDPC_N	0.82	166334.30	59308.57
HT-74	AUDPC_N	0.88	281812.70	66853.40
HT-96	AUDPC_N	0.83	144762.50	47844.46
HT-18	AUDPC_P	0.67	18020.60	11743.40
HT-44	AUDPC_P	0.77	11047.91	5128.44
HT-53	AUDPC_P	0.73	30859.82	16778.84
HT-74	AUDPC_P	0.79	60558.65	25641.09
HT-96	AUDPC_P	0.65	4798.34	3326.06

Genotypic variance (*V*_*g*_) and mean prediction error variance (PEV) are also shown

#### Correlation between AUDPC_N and AUDPC_P phenotypes across isolates

**Fig. 4:**
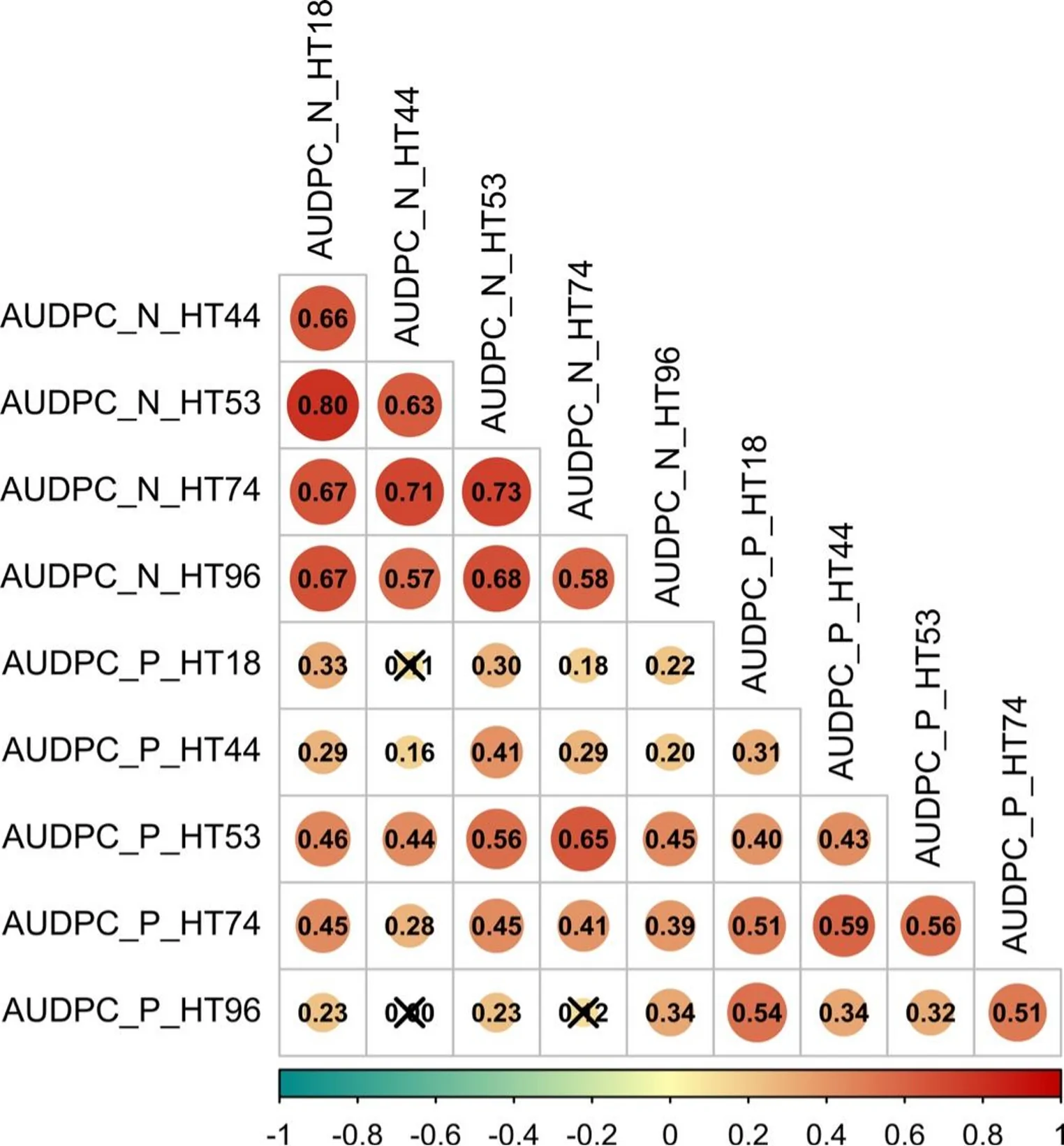
Spearman correlation matrix for AUDPC_N and AUDPC_P across five *Zymoseptoria tritici* isolates. Pairwise associations among isolate-specific genotype BLUEs were quantified using Spearman’s rank correlation coefficient (*ρ*). *P*-values for the 45 pairwise tests were adjusted using the Benjamini–Hochberg procedure. Black crosses denote correlations that were not significant after adjustment (adjusted *p* ≥ 0.05)

Spearman rank correlation analysis using isolate-specific genotype BLUEs revealed moderate positive correlations among necrosis traits across isolates (*ρ* = 0.57–0.80, *p* < 0.05) (Fig. 4). Correlations among AUDPC_P BLUEs were weaker but consistently positive (*ρ* = 0.32–0.59), while correlations between necrosis and pycnidia were generally weaker still (*ρ* = 0.18–0.46). Statistical significance after Benjamini–Hochberg correction is indicated in Fig. 4. These patterns show that, although necrosis responses were moderately consistent across isolates, substantial isolate-specific variation remained, and associations between necrosis and pycnidia development were weaker. The partial decoupling of necrosis and pycnidia was particularly evident for some isolate-trait combinations, supporting the conclusion that these traits represent partly independent components of the host response to STB.

### Genotypic analysis

#### Genomic variation, population structure, and linkage disequilibrium

Analysis of SNP marker distribution across the three wheat subgenomes revealed a clear disparity in polymorphism (**Supplementary Table S3**). The B-subgenome exhibited the highest marker density, averaging 486.1 SNPs per chromosome, whereas the D-subgenome showed substantially lower variation, averaging 191.1 SNPs per chromosome. This pattern is consistent with the reduced genetic diversity of the D-subgenome resulting from the evolutionary bottleneck associated with hexaploid wheat formation. However, the lower proportion of retained D-subgenome markers relative to their representation on the Axiom 35K Wheat Breeders’ array suggests that ascertainment bias in marker design may also have contributed to this disparity (**Supplementary Table S3**).

**Fig. 5:**
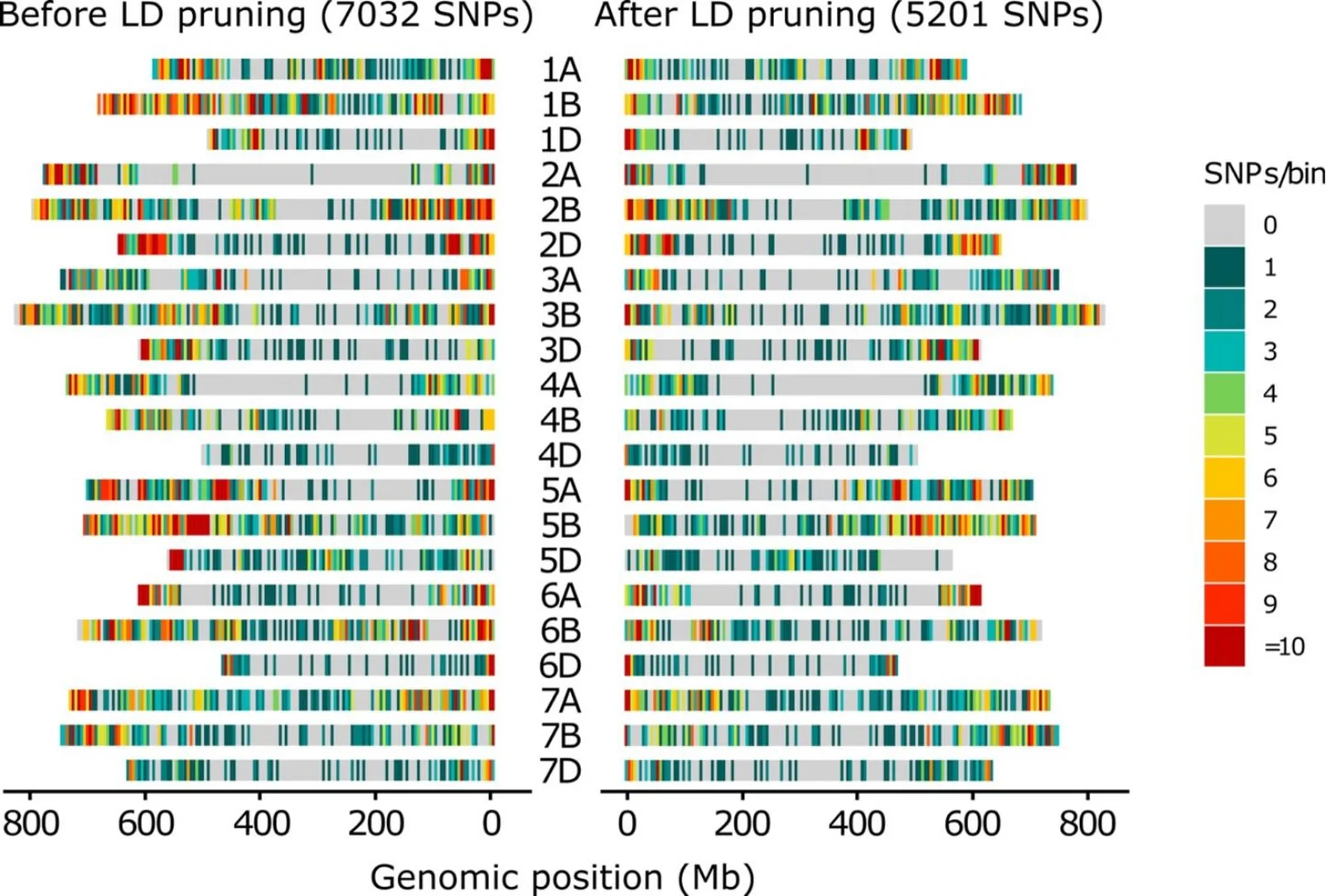
SNP distribution across the wheat genome before and after LD pruning. SNP genomic positions are shown for the full marker set (left) and the LD-pruned marker set (right), in which redundant SNPs in perfect linkage disequilibrium (*r²* = 1) were removed

Marker distribution across the physical length of all 21 chromosomes indicated sufficient density for downstream LD analysis and GWAS. Prior to genetic analyses, markers were pruned to minimise redundancy by removing SNPs in perfect LD (*r²* = 1). Because LD-based pruning filters markers on the basis of statistical correlation rather than physical position, the resulting pruned dataset does not directly represent physical genome coverage. Consequently, chromosome and subgenome coverage were evaluated using the unpruned dataset, whereas the LD-pruned dataset was used to reduce marker redundancy for downstream population structure and association analyses (Fig. 5).

**Fig. 6:**
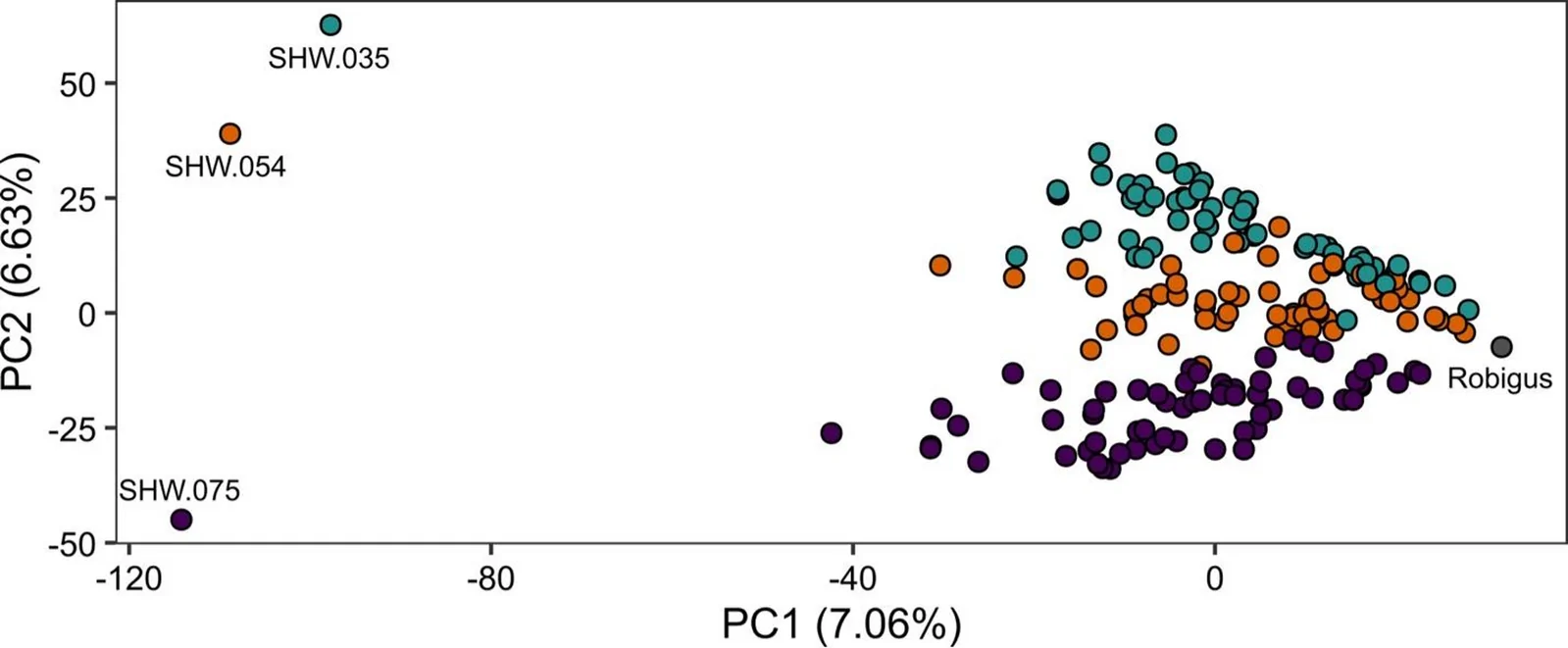
Principal component analysis of genetic variation within the NAM population. SHW.035 and its derived NAM.144 lines are shown in teal, SHW.054 and NAM.212 in orange, SHW.075 and NAM.233 in purple, and Robigus in grey

PCA revealed clear population structure within the NAM panel, driven primarily by the synthetic founders (Fig. 6). PC1 and PC2 together explained 13.69% of the total genetic variation (PC1 = 7.06%, PC2 = 6.63%). The four founders (SHW.035, SHW.054, SHW.075, and Robigus) formed distinct genetic groupings. Progeny derived from SHW.035 and SHW.054 formed partially overlapping clusters, consistent with their shared tetraploid donor (Table 1). Subgenome-specific comparison of founder SNPs showed that SHW.035 and SHW.054 exhibited relatively little differentiation across the A- and B-subgenomes but extensive differentiation across the D-subgenome (**Supplementary Fig. S7**). Differentiating SNPs between these founders were distributed across all D-subgenome chromosomes rather than being confined to specific regions, consistent with their distinct *Ae. tauschii* lineage donors (L1 for SHW.035 and L2 for SHW.054). In contrast, SHW.075 displayed broader genome-wide differentiation, reflecting divergence in both tetraploid and D-subgenome ancestry. As expected for backcross-derived families, NAM lines clustered closer to Robigus than to their SHW parents.

#### Genome-wide association study

GWAS was conducted to identify resistance-associated genomic regions in the NAM population. Analyses used isolate-specific BLUEs for AUDPC_N and AUDPC_P across the five isolates (**Supplementary Table S4**).

Genome-wide LD decay analysis showed that *r*^*2*^ declined to 0.2 at approximately 50 Mb (**Supplementary Fig. S6**). This relatively slow LD decay is consistent with the backcross-derived nature of the population, limited historical recombination, and relatedness among lines. The estimated LD-decay distance was used to distinguish independent associated regions, with the most significant SNP within each region designated as the peak marker. Across traits and isolates, several significant resistance-associated regions were identified (Table 3; **Supplementary Table S5**).

**Table 3:** Significant resistance-associated QTLs detected for necrosis (AUDPC_N) and pycnidia development (AUDPC_P) following inoculation with five *Zymoseptoria tritici* isolates in the three-family wheat NAM population

QTL	Trait	Isolate	Significance threshold	Marker	Chr	Position (Mb)	-log_10_(*p*)	Corrected effect	Corrected Var. (%)
*qSTB-3D.1*	AUDPC_N	HT-18	4.4	AX-95,090,074	3D	609.21	5.62	-395.24	12.32
*qSTB-6D.1*	AUDPC_N	HT-18	4.4	AX-95,175,637	6D	27.42	4.94	-198.90	8.68
*qSTB-3 A.1*	AUDPC_N	HT-53	4.29	AX-94,766,803	3 A	26.04	5.04	-334.58	17.04
*qSTB-3D.1*	AUDPC_N	HT-53	4.29	AX-95,090,074	3D	609.21	5.01	-374.94	18.80
*qSTB-3D.1*	AUDPC_N	HT-74	4.42	AX-95,090,074	3D	609.21	5.73	-329.67	15.60
*qSTB-3D.1*	AUDPC_N	HT-96	4.29	AX-95,090,074	3D	609.21	6.71	-382.16	15.94
*qSTB-1B.2*	AUDPC_P	HT-96	4.88	AX-95,193,381	1B	647.23	4.99	101.60	10.53

The table lists the chromosome (Chr), physical position (Mb), and −log10*(p)* value of the peak SNP for each resistance-associated QTL. Genome-wide significance thresholds were determined separately for each isolate and trait using 1,000 permutations at *α* = 0.05. Allelic effects are additive marker-effect estimates from the GWASpoly association analysis and are expressed on the same scale as the isolate-specific AUDPC BLUEs. The Robigus allele was used as the reference; negative values indicate reduced disease associated with the alternative allele, whereas positive values indicate increased disease. ‘Corrected Var. (%)’ indicates the proportion of phenotypic variance attributable to each QTL when significant QTLs for the corresponding isolate and trait were fitted jointly in the final QTL model

#### Genome-wide association mapping for necrosis

**Fig. 7:**
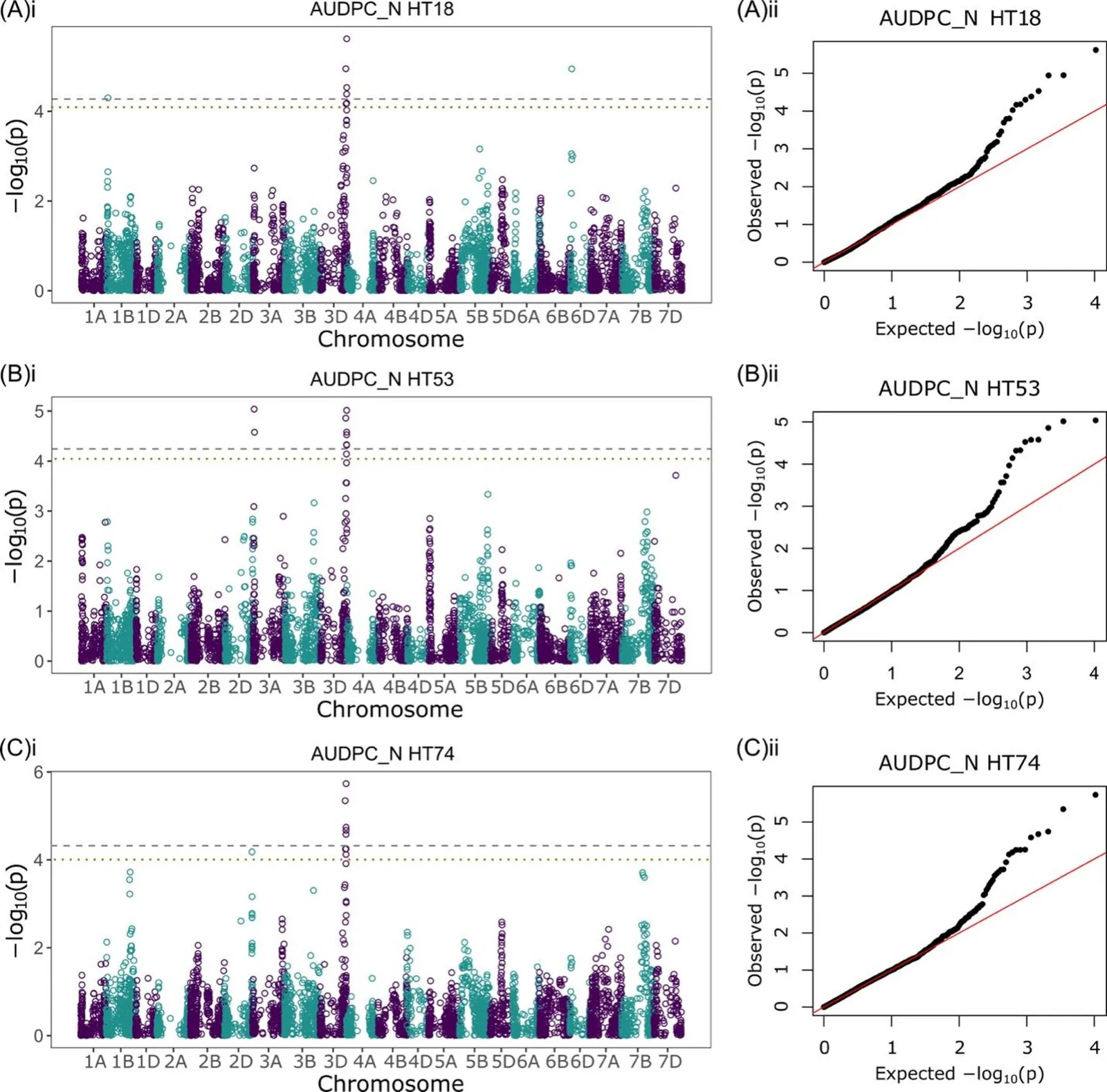
GWAS for AUDPC_N in the NAM population. Manhattan plots (**Ai–Ci**) and corresponding quantile-quantile plots (Aii–Cii) for GWAS of AUDPC_N following inoculation with *Z. tritici* isolates: (**A**) HT-18, (**B**) HT-53, and (**C**) HT-74. The dashed grey horizontal line indicates the genome-wide significance threshold determined using 1,000 permutations at *α* = 0.05 for each isolate, while the dotted yellow line denotes the false discovery rate threshold at *q* = 0.05

A highly reproducible candidate resistance-associated interval on chromosome arm 3DL, designated *qSTB-3D.1*, was detected in response to four of the five tested isolates: HT-18, HT-53, HT-74, and HT-96 (Table 3; Fig. 7; **Supplementary Fig. S8**). No significant association at this region was detected in response to HT-44 (**Supplementary Fig. S8**). In all four responsive isolate analyses, the SHW-derived allele was associated with reduced AUDPC_N.

Segregation of the resistance-associated allele was family specific: a subset of NAM.144 lines carried the favourable allele, whereas the other two NAM families were fixed for the Robigus allele, consistent with SHW.035 being the donor of the favourable haplotype at this interval. Across all four responsive isolates, significant associations mapped to a common genomic interval spanning 587.09–610.79 Mb on chromosome 3D. The same peak SNP, AX-95,090,074 at 609.21 Mb, was identified for all four isolates. Because of the extended LD within this backcross-derived NAM population, these associations define a broad 23.7 Mb candidate resistance-associated interval and do not resolve the underlying causal gene.

A total of 425 high-confidence genes were identified within the *qSTB-3D.1* interval (**Supplementary Table S6**). The interval contains several classes of genes with potential roles in plant immunity. A cluster of seven CRK genes between 590.04 and 590.31 Mb includes the cloned STB resistance gene *Stb16q* (*TraesCS3D02G500800*) (Tabib Ghaffary et al. 2012; Saintenac et al. 2021). However, the strongest association at ~ 609 Mb lies approximately 19 Mb distal to this CRK cluster and close to the WAK gene (*TraesCS3D02G533100*) at 608.92 Mb. Additional WAKs, RLKs, NLRs, and RLPs are also present within the interval (Table 4; **Supplementary Table S6**). The available mapping resolution therefore does not allow *Stb16q*, the WAK near the association peak, or other linked genes to be distinguished as the causal basis of *qSTB-3D.1*.

**Table 4:** Candidate resistance genes identified within resistance-associated QTL intervals

QTL	Start position	End position	Window size	CRK	WAK	LecRK	LRR-RLK	NLR	Kinase	RLP
*qSTB-1B.1*	28,560,996	32,769,214	4.21	0	1	0	0	3	0	0
*qSTB-1B.2*	646,172,198	651,275,182	5.10	0	0	0	0	0	0	0
*qSTB-2D.1*	641,106,101	643,193,656	2.09	1	0	0	0	0	2	0
*qSTB-3 A.1*	24,057,407	35,463,415	11.41	1	10	0	0	1	1	4
*qSTB-3D.1*	587,096,644	610,795,248	23.70	7	6	0	3	12	6	13
*qSTB-6D.1*	25,615,199	34,974,332	9.36	0	3	0	1	1	1	5

Physical genomic coordinates and interval sizes (Mb) are based on the IWGSC RefSeq v.1.1 assembly. Candidate genes are categorised by functional class: CRK, cysteine-rich receptor-like kinase; WAK, wall-associated kinase; LecRLKs, lectin receptor-like kinase; LRR-RLK, leucine-rich repeat receptor-like kinase; NLR, nucleotide-binding leucine-rich repeat; Kinase, unspecified kinase; RLP, receptor-like protein. Gene-class annotations identify plausible candidates within associated intervals but do not imply causal identity

#### Isolate-specific resistance-associated intervals for necrosis

In addition to *qSTB-3D.1*, several resistance-associated intervals were detected only in response to individual isolates (Table 3; **Supplementary Table S5;** Fig. 7; **Supplementary Fig. S8**). *qSTB-6D.1* and *qSTB-3 A.1* exceeded the permutation-derived significance threshold, whereas *qSTB-1B.1* and *qSTB-2D.1* met the FDR threshold but not the permutation threshold.

Two additional associations were identified in response to HT-18 (Fig. 7A). The first, *qSTB-1B.1*, mapped to 4.21-Mb interval on chromosome arm 1BS (28.56–32.77 Mb), and the resistance-associated allele was restricted to the SHW.075-derived family. Candidate gene analysis identified a WAK gene (*TraesCS1B02G050100*) at 29.92 Mb within this interval (Table 4; **Supplementary Table S7**). The second HT-18-specific association, *qSTB-6D.1*, mapped to chromosome 6D at ~ 27 Mb. The favourable allele was present in all three SHW founders. The interval contains several plausible defence-related candidates including the WAK gene (*TraesCS6D02G056600*) at 27.06 Mb (Table 4; **Supplementary Table S8**).

A resistance-associated interval on chromosome 3A, *qSTB-3A.1*, was detected only in response to HT-53 (Fig. 7B, **Supplementary Fig. S8**). The approximately 11.41 Mb interval was contributed by SHW.075 and contains ten WAK genes, including the cloned resistance gene *Stb6* (*TraesCS3A02G049500*) (Saintenac et al. 2018) (Table 4; **Supplementary Table S9**). The mapping resolution does not distinguish allelic variation at *Stb6* from the effects of other linked genes within this WAK-rich interval.

Finally, *qSTB-2D.1* was detected in response to HT-74 (Fig. 7C). This relatively narrow 2.09 Mb interval spans 641.11–643.19 Mb. The resistance-associated allele was present in all three SHW founders. A CRK-encoding gene (*TraesCS2D02G579600*) and two additional kinase domain-encoding genes were identified within the interval (Table 4; **Supplementary Table S10**).

#### GWAS for pycnidia development

**Fig. 8:**
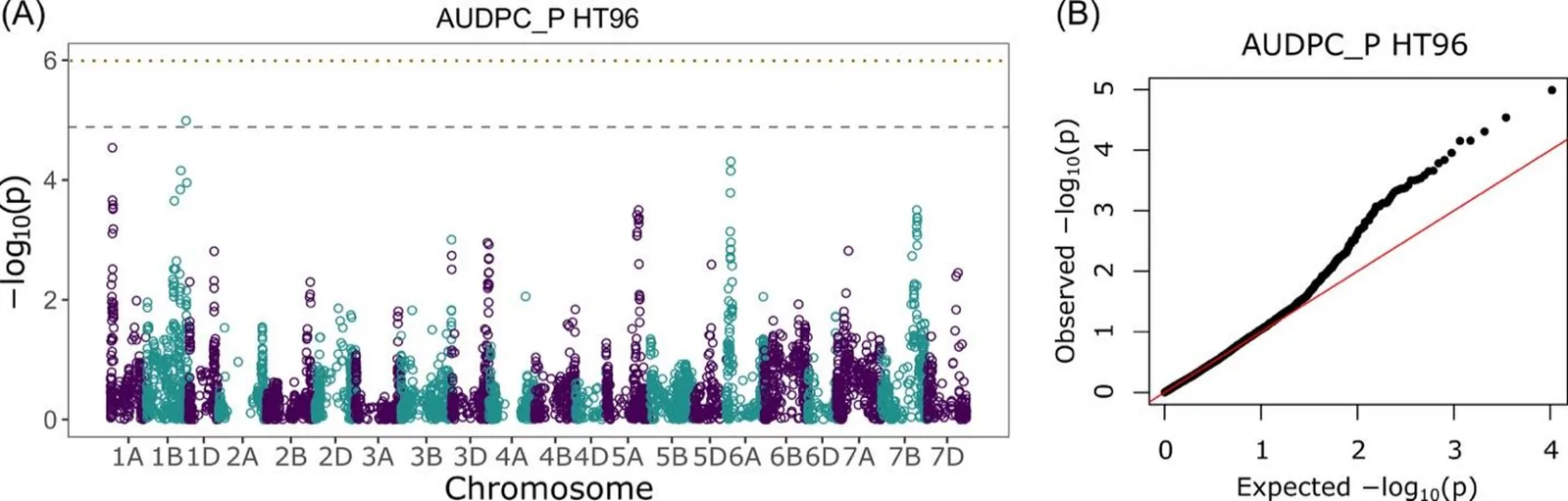
GWAS for AUDPC_P in the NAM population following inoculation with *Zymoseptoria tritici* isolate HT-96. Manhattan plot (**A**) and corresponding quantile–quantile plot (**B**). The dashed grey horizontal line indicates the genome-wide significance threshold determined using 1,000 permutations at *α* = 0.05 for each isolate, while the dotted yellow line denotes the false discovery rate threshold at *q* = 0.05

In contrast to necrosis, GWAS identified few significant associations for pycnidia development. A single resistance-associated region on chromosome arm 1BL, *qSTB-1B.2*, was detected in response to HT-96 at ~ 647.23 Mb (Table 3; Fig. 8). The resistance-associated allele was contributed by Robigus. No obvious immune receptor-like genes were identified within the corresponding interval (**Supplementary Table S11**). No significant associations were detected for the remaining four isolates (**Supplementary Fig. S9**).

### Segregation of resistance phenotypes in the SHW.BC.144.12.1.4.1 × Robigus BC_2_F_2_ population

**Fig. 9:**
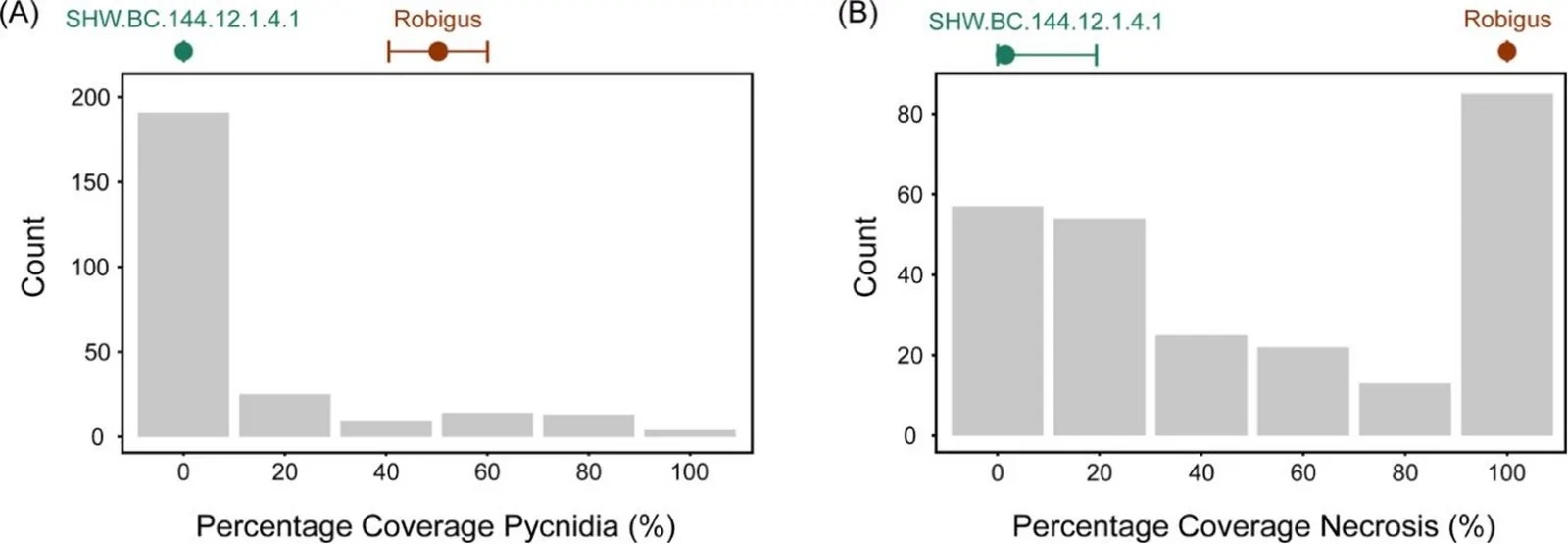
Phenotypic distributions of pycnidia and necrosis in the SHW.BC.144.12.1.4.1 × Robigus BC_2_F_2_ population. Histograms show percentage leaf coverage by pycnidia (**A**) and necrosis (**B**) scored at 29-dpi following inoculation with *Zymoseptoria tritici* isolate HT-74. Counts indicate the number of BC_2_F_2_ individuals in each phenotypic class. Parental phenotypes (SHW.BC.144.12.1.4.1, teal; Robigus, orange) are shown above each histogram, with points indicating median parental scores and vertical bars representing the range across replicate plants

The BC_2_F_2_ population derived from the resistant SHW.035-derived NAM line SHW.BC.144.12.1.4.1 and Robigus was assessed for resistance to *Z. tritici* isolate HT-74. Percentage leaf coverage by pycnidia and by necrosis was scored at 29-dpi (Fig. 9).

The distribution of pycnidia coverage was strongly skewed towards resistance (Fig. 9A). Most individuals showed no visible pycnidia, resembling the resistant parent, whereas fewer individuals exhibited moderate or high levels. A small number showed greater pycnidia coverage than Robigus. Because scoring was performed in 20-percentage-point increments, individuals with 0% pycnidia coverage were classified as resistant and those with ≥ 20% coverage as susceptible. The observed segregation of 191 resistant and 65 susceptible BC₂F₂ individuals did not differ from a 3:1 expectation (*χ²* = 0.021, *p* = 0.885), consistent with segregation of a single dominant resistance factor.

For necrosis, individuals with 0–20% leaf coverage were classified as resistant and those with ≥ 40% coverage as susceptible (Fig. 9B). The observed segregation did not conform to either a 3:1 (*χ*² = 136.69, *df* = 1, *p* < 2.2 × 10⁻¹⁶) or a 1:3 (*χ*² = 46.02, *df* = 1, *p* = 1.17 × 10⁻¹¹) single gene expectation, indicating more complex genetic control of necrosis in this population.

## Discussion

This study used a SHW-derived NAM population to dissect resistance to *Z. tritici* across multiple isolates differing in virulence. By integrating multi-isolate phenotyping with genetic mapping, the study identified resistance-associated regions with effects across several isolates as well as associations detected only with individual isolates. Importantly, the results indicate that genetic factors limiting pathogen reproduction are at least partly distinct from those influencing host tissue damage.

### Isolate-specific virulence and host response variation

The five *Z. tritici* isolates used in this study produced distinct disease responses across contrasting wheat genetic backgrounds (Fig. 2). Differences were particularly apparent for pycnidia development, with HT-74 generally producing greater sporulation and HT-96 lower sporulation, whereas necrosis responses depended more strongly on host genetic background. Such variation is consistent with the extensive genetic and effector diversity characteristic of natural *Z. tritici* populations (McDonald et al. 2022).

A striking feature of the SHW-derived material was the frequent partial uncoupling of necrosis and pycnidia development (Figs. 3 and 4). Many genotypes showed strong suppression of pycnidia despite developing visible necrosis, and correlations between AUDPC_N and AUDPC_P were generally weaker than correlations among the same disease component across isolates. Similar phenotypes, in which fungal ingress or lesion development occurs but subsequent pathogen growth or reproduction is restricted, have been described in wheat lines carrying several *Stb* resistance genes, including *Stb3*, *Stb5* and *Stb6* (Tidd et al. 2023). Fungal development on resistant wheat can also persist at early stages without progressing efficiently to reproduction (Fones et al. 2023).

Several complementary mechanisms could explain the partial uncoupling observed here. Host recognition following fungal entry could activate local defence and cell death while restricting subsequent apoplastic proliferation or the transition to pycnidia formation. Alternatively, necrosis and fungal reproduction may be influenced by partly independent host loci or physiological pathways. These possibilities are not mutually exclusive, and visual symptom scoring alone cannot distinguish among them. Microscopy, fungal biomass measurements and temporal analysis of defence responses would help determine more precisely at which stage pathogen development is restricted.

From an epidemiological perspective, suppression of pycnidia is particularly important because it directly limits production of secondary inoculum and subsequent spread within the crop canopy. The segregation analysis in the SHW.BC.144.12.1.4.1 × Robigus BC₂F₂ population provides further support for differences in the genetic control of the two disease components (Fig. 9). Pycnidia suppression segregated in a 3:1 ratio consistent with a single dominant resistance factor in this cross, whereas necrosis did not conform to a simple single-gene model.

Together, these results suggest that resistance in this SHW-derived material may involve a major factor that strongly restricts pathogen reproduction, with additional genetic effects influencing the extent of host tissue damage. One possible explanation is receptor-mediated pathogen recognition, potentially involving WAKs, RLKs or other immune receptors that activate defence responses sufficient to restrict fungal proliferation or pycnidia formation while still allowing localised necrosis. Such mechanisms could contribute to the partial genetic decoupling of pathogen reproduction and host tissue damage observed here. More broadly, combining a strong major resistance factor with additional quantitative resistance has been proposed as one approach to improving resistance durability in other plant–pathogen systems (Palloix et al. 2009).

### Candidate multi-isolate resistance-associated interval on chromosome 3D

The most reproducible GWAS signal in this study was *qSTB-3D.1*, which was detected in response to four of the five *Z. tritici* isolates tested (Table 3; Fig. 7; **Supplementary Fig. S8**). The resistance-associated allele was contributed by SHW.035, whose D-genome donor was *Ae. tauschii* accession WX224, and was not detected in the other two NAM families. The repeated detection of this resistance-associated interval in four of the five isolates indicates that it contributes resistance to multiple pathogen genotypes, although the absence of a significant association with HT-44 indicates that its effect is not universal.

The 587.09–610.79 Mb *qSTB-3D.1* interval encompasses a cluster of CRK genes that includes *Stb16q*, a cloned STB resistance gene first identified in the SHW line M3 (W-7976) developed by CIMMYT (Tabib Ghaffary et al. 2012; Saintenac et al. 2021). *Stb16q* was considered a particularly relevant candidate because it is a cloned and functionally characterised STB resistance gene located within the associated interval, was originally identified in synthetic wheat, and confers resistance to diverse *Z. tritici* isolates. These prior biological observations, however, do not provide stronger positional support for *Stb16q* than for other genes within the interval.

The current data do not allow *qSTB-3D.1* to be assigned to *Stb16q*. The strongest association across the four responsive isolates was at 609.21 Mb, approximately 19 Mb distal to the *Stb16q*-containing CRK cluster and close to the WAK gene *TraesCS3D02G533100* at 608.92 Mb (Table 4; **Supplementary Table S6**). WAKs are also biologically plausible candidates because members of this receptor class, including *Stb6*, mediate resistance to *Z. tritici* (Saintenac et al. 2018). Thus, while *Stb16q* has strong prior biological support, the WAK near the association peak is at least as plausible on positional grounds. The extensive LD in this backcross-derived NAM population means that the *Stb16q* region, the WAK-associated region around 609 Mb and other linked genes within the 23.7 Mb interval cannot presently be distinguished.

Resistance-associated regions spanning parts of this 3DL have also been reported in independent studies. Odilbekov et al. (2019) identified an STB resistance QTL spanning approximately 593.4–614.4 Mb and noted uncertainty regarding its relationship to *Stb16q*. More recently, Binalf et al. (2024) also identified association with STB resistance in the region around 609 Mb. Together with the results presented here, these studies emphasise that the broader 3DL region may contain closely linked resistance determinants that are difficult to resolve in populations with extended LD.

Analysis using published *Stb16q* diagnostic markers or targeted sequencing could establish whether SHW.035 carries the previously characterised *Stb16q* resistance allele or distinct allelic variation at this gene. However, such information alone would not demonstrate that *Stb16q* is responsible for the *qSTB-3D.1* association because the more distal 609 Mb region remains genetically linked in the present population. Fine mapping using informative recombinants across the interval, followed by targeted sequence and functional analyses, will therefore be required to determine whether *qSTB-3D.1* reflects *Stb16q*, the WAK-associated region near 609 Mb, or another linked resistance factor.

The emergence of *Z. tritici* isolates virulent towards *Stb16q-*containing wheat varieties (such as Cellule) in European populations further underlines the importance of resolving the genetic basis of this interval and evaluating its effectiveness against more recently sampled field isolates (Kildea et al. 2020; Orellana-Torrejon et al. 2022).

### Isolate-specific resistance-associated intervals on chromosomes 1B, 2D, 3A and 6D

In addition to *qSTB-3D.*1, several resistance-associated intervals were detected only in response to individual isolates (Table 3; Fig. 7; **Supplementary Fig. S8**). These isolate-specific associations could reflect interactions between wheat resistance factors and isolate-specific pathogen determinants, including possible gene-for-gene relationships, although functional validation will be required to establish the underlying mechanisms.

*qSTB-1B.1*, which met the FDR but not the permutation-derived significance threshold, detected in response to HT-18, maps to 1BS within a chromosome region known to harbour several STB resistance genes, including *Stb2* (Liu et al. 2013), *Stb11* (Chartrain et al. 2005b), and several *StbWW* loci (Raman et al. 2009). However, the interval identified here lies outside the previously defined major resistance clusters and the *QStb.wai.1B.1* region (Yang et al. 2022), suggesting that *qSTB-1B.1* may represent a distinct resistance-associated interval or allelic variation within this broader resistance gene-rich region. A WAK gene, *TraesCS1B02G050100*, occurs within the associated interval (Table 4; **Supplementary Table S7**), but functional validation will be required before this or another candidate can be assigned as causal.

The second HT-18-specific association, *qSTB-6D.1*, partially overlaps the reported position of *Stb18*, an isolate-specific resistance gene (Tabib Ghaffary et al. 2011). However, the peak association falls outside the currently defined *Stb18* interval, leaving open the possibility that the signal reflects *Stb18*, a closely linked resistance factor, or another locus within the associated region. Notably, the other isolates used in this study were virulent on Balance, which carries *Stb6* and *Stb18* (Tidd et al. 2023), consistent with the isolate-dependent nature of the HT-18 response. Several defence-related candidate genes are present within the interval (Table 4; **Supplementary Table S8**).

On chromosome 3A, *qSTB-3A.1* was detected exclusively in response to HT-53 and was contributed by SHW.075 (Table 3; Fig. 7B). This region overlaps the location of *Stb6*, which encodes a WAK mediating gene-for-gene resistance (Brading et al. 2002; Saintenac et al. 2018). Although *Stb6* is present in many European varieties, including Robigus (Chartrain et al. 2005a; Goudemand et al. 2013), the resistance-associated allele at *qSTB-3A.1* was contributed by SHW.075. Allelic variation at the *Stb6* locus is therefore one plausible explanation, but a tightly linked resistance gene within the same WAK-rich region cannot be excluded (Table 4; **Supplementary Table S9**).

*qSTB-2D.1* was also detected only at the FDR threshold in response to isolate HT-74 and is of particular interest because no major named *Stb* gene has been reported on chromosome 2D. This interval overlaps previously reported STB resistance QTLs identified in synthetic-derived germplasm (Naz et al. 2015; Riaz et al. 2020), suggesting that chromosome 2D may represent a recurrent source of STB resistance variation. The interval contains a CRK-encoding gene (*TraesCS2D02G579600*) together with two additional kinase-domain genes (Table 4; **Supplementary Table S10**). Nevertheless, until independent genetic and functional validation is available, *qSTB-2D.1* should be regarded as a resistance-associated genomic interval rather than a confirmed novel resistance locus.

Collectively, these isolate-specific associations identify several genomic regions that warrant further validation using the corresponding *Z. tritici* isolates. Confirmation in independent segregating populations will be particularly important for determining whether these intervals represent distinct resistance factors or allelic variation at, or linkage to, previously characterised *Stb* genes.

### Genetic architecture of pycnidia resistance

In contrast to necrosis severity, GWAS identified very few loci associated with AUDPC_P. Only one significant association, *qSTB-1B.2*, was detected in response to HT-96 (Table 3; Fig. 8), and no significant associations were identified for the other four isolates (**Supplementary Fig. S9**). This occurred despite moderate-to-high broad-sense heritability for AUDPC_P (Table 2).

One likely explanation is the limited phenotypic variation available for association mapping because pycnidia development was strongly suppressed across much of the SHW-derived material (Figs. 2 and 3). In addition, strong pycnidia resistance alleles may be fixed or present at high frequency within individual families, or shared among several SHW-derived lineages. In a NAM population, loci showing little within-family segregation or limited allelic contrast across the population can be difficult to detect even when they have substantial phenotypic effects (Yu et al. 2008; McMullen et al. 2009; Korte and Farlow 2013).

The resolution of the phenotyping scale may have further reduced sensitivity to smaller genetic effects. Percentage leaf coverage by pycnidia was scored in 20-percentage-point increments, producing a discrete phenotype that may compress variation, particularly where many genotypes cluster near the resistant end of the distribution. These explanations are complementary rather than mutually exclusive: restricted phenotypic variance, high or fixed frequencies of favourable alleles, shared resistance alleles among SHW founders and the resolution of visual scoring could all have contributed to reduced GWAS power for pycnidia resistance.

The segregation observed in the SHW.BC.144.12.1.4.1 × Robigus BC₂F₂ population further illustrates why targeted biparental populations may be useful for dissecting this trait. Pycnidia suppression segregated in a 3:1 ratio consistent with a single dominant resistance factor in this specific cross (Fig. 9), despite the absence of a corresponding major AUDPC_P association in the wider NAM GWAS. This Mendelian segregation pattern cannot be assumed to extend across the whole NAM population, where the relevant favourable allele may be fixed within individual families or occur at high frequency across multiple SHW-derived lineages.

The only AUDPC_P association detected by GWAS, *qSTB-1B.2*, did not contain an obvious annotated immune receptor-like candidate (Table 4; **Supplementary Table S11**). Further work will therefore be required to determine whether this association represents a relatively small background-dependent effect or variation in a gene not readily identifiable from current functional annotation.

### Limitations and future directions

While this study provides a detailed analysis of STB resistance in a SHW-derived NAM population, several limitations should be acknowledged. First, the GWAS population comprised 171 phenotyped and genotyped NAM lines. Although the structured population and intensive multi-isolate phenotyping enabled reproducible associations to be detected, this population size limits power for detecting small effect or low frequency alleles, particularly where favourable alleles segregate in only one family. The phenotyping design was also unbalanced across experimental batches and did not use formal randomisation. Mixed models explicitly accounted for batch and relevant batch interaction effects, although residual positional effects cannot be excluded.

Second, disease phenotyping relied on visual scoring using discrete 20-percentage-point intervals. Although this approach was sufficient to capture major differences in disease severity, it is inherently subjective and may reduce sensitivity for subtle phenotypic variation associated with minor genetic effects. Image-based phenotyping provides an opportunity to address this limitation through continuous, higher-resolution quantification of necrotic and sporulating tissue on living leaves over time. Such approaches should improve both the power and precision of future genetic analyses.

Third, all phenotyping was conducted at the seedling stage under controlled conditions. Resistance expressed at the adult-plant stage may involve additional loci and environmental interactions. Validation of the resistance-associated regions identified here under field conditions, particularly under high disease pressure and across multiple environments, will therefore be important to establish their breeding relevance and stability.

The causal basis of *qSTB-3D.1* also remains unresolved because of the broad 23.7 Mb interval and extended LD. Fine mapping with informative recombinants, together with targeted sequence and functional analyses, will be required to distinguish *Stb16q* from the WAK-associated region near 609 Mb and other linked candidates. Given reports of *Z. tritici* isolates virulent towards *Stb16q* in European populations, testing the effectiveness of the underlying resistance factor against contemporary field isolates will also be important for assessing its potential durability and deployment in breeding programmes.

The isolate-specific resistance-associated intervals on chromosomes 1B, 2D, 3A and 6D likewise represent promising targets for future validation and functional characterisation. Testing these regions in appropriate segregating populations with the corresponding *Z. tritici* isolates, followed by fine mapping and candidate-gene analysis, should help establish their genetic basis and potential value for breeding.

The SHW.BC.144.12.1.4.1 × Robigus BC₂F₂ population provides a particularly useful resource for further dissection of resistance inherited from SHW.035. Targeted biparental analysis should help resolve resistance factors that are difficult to distinguish within the wider NAM population, while the backcross-derived nature of this material may facilitate subsequent deployment in adapted wheat backgrounds.

Overall, this study shows that resistance to STB in the investigated SHW-derived NAM population has a complex genetic basis, with resistance-associated effects differing in their breadth and potentially acting through distinct mechanisms. Higher-resolution phenotyping, field validation, and targeted fine mapping should allow these resistance-associated intervals to be resolved more fully and their breeding value to be assessed. More broadly, the multi-isolate GWAS strategy enabled resistance effective across several pathogen genotypes to be distinguished from isolate-specific genetic effects, highlighting the value of phenotyping with diverse pathogen isolates for dissecting complex STB resistance.

## Supplementary Information

Below is the link to the electronic supplementary material.

Supplementary Material 1

Supplementary Material 2

## Data Availability

All the data supporting the findings of this study are available within the paper and within its Supporting Information.
