# Supplementary material for: Multi-isolate GWAS identifies broad-spectrum and isolate-specific resistance-associated loci for Septoria tritici blotch in synthetic hexaploid wheat
Source: Theor Appl Genet. 2026 Oct 20;139(11):309. doi: 10.1007/s00122-026-05423-2 (PMC13683512; doi:10.1007/s00122-026-05423-2)

Supplementary Figure S1. Histograms showing the distributions of heterozygosity (%) and missing data (NoCall rate, %) across markers and genotypes before and after quality filtering. Panels (A-D) show marker-level distributions: heterozygosity before filtering (A) and after filtering (B), and NoCall rate before filtering (C) and after filtering (D). Panels (E-H) show genotype-level distributions: heterozygosity before filtering (E) and after filtering (F), and NoCall rate before filtering (G) and after filtering (H). Dotted blue lines indicate the applied filtering threshold of 7%. Filtering removed markers and genotypes exceeding this threshold, thereby improving data quality for downstream analyses.

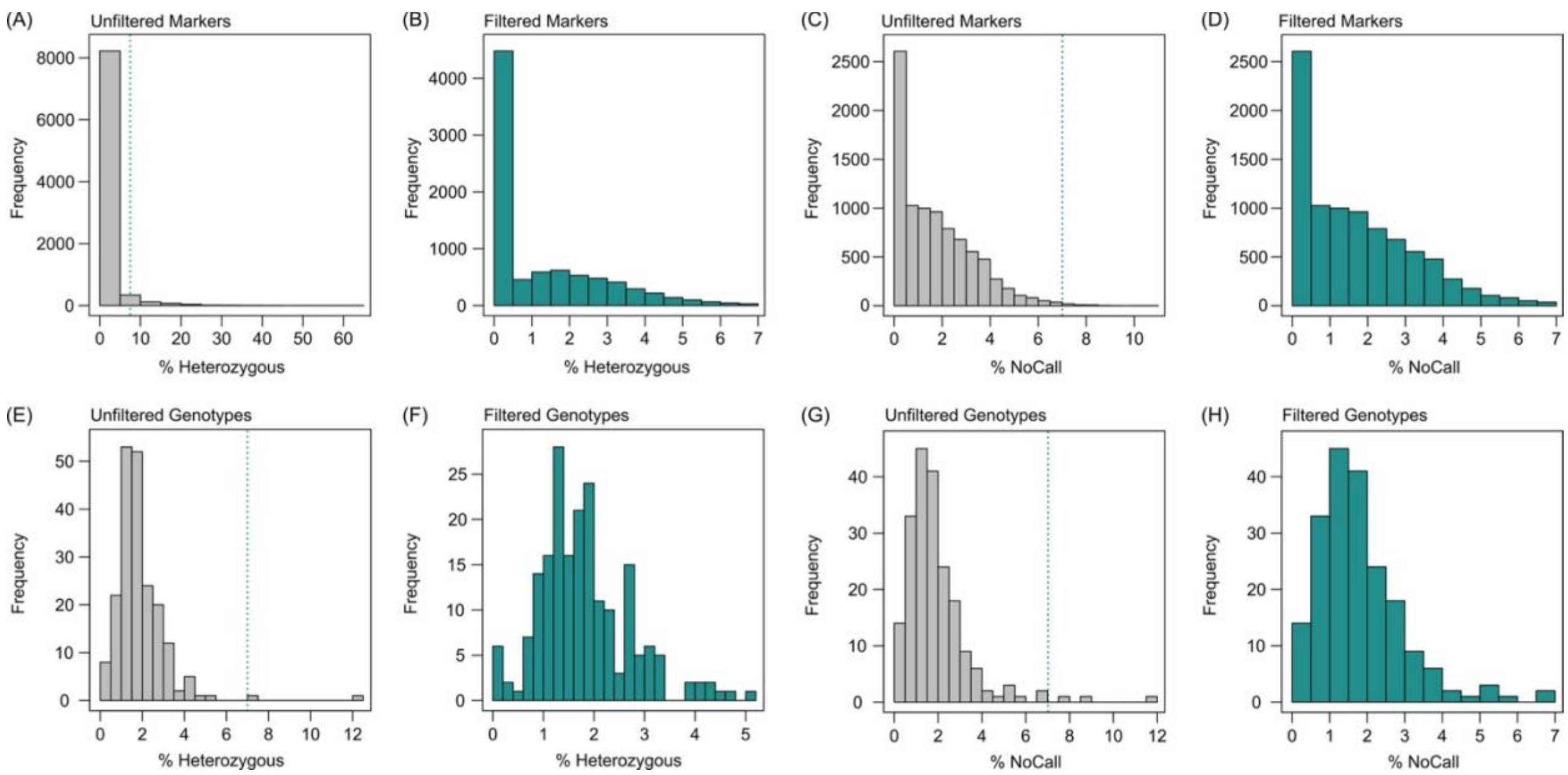

Supplementary Figure S2. Principal coordinate analysis (PCoA) of genotype data used to identify potential outlier lines within the nested association mapping (NAM) population. PCoA plots are shown separately for each NAM family: (A) NAM.144, (B) NAM.212, and (C) NAM.233. Points represent individual NAM lines and are coloured according to their backcross lineage, with lines originating from the same backcross stream shown in the same colour. The corresponding parental lines are shown in black and labelled.

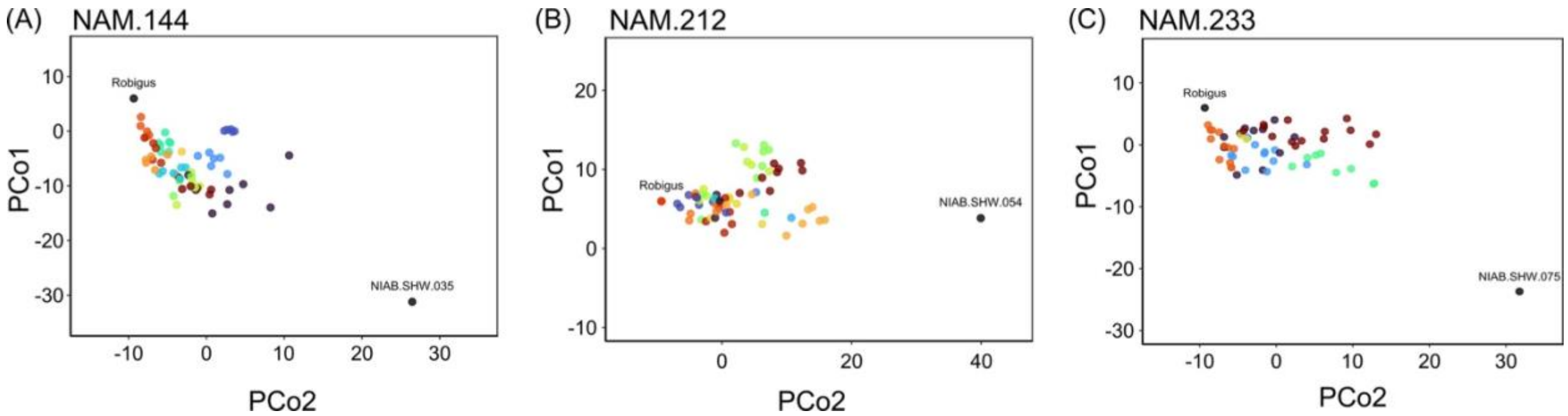

Supplementary Figure S3. Linkage disequilibrium (LD) between SNP markers on each chromosome prior to marker repositioning. LD was calculated as pairwise correlation (R^2) among 5,327 previously anchored markers within each chromosome.

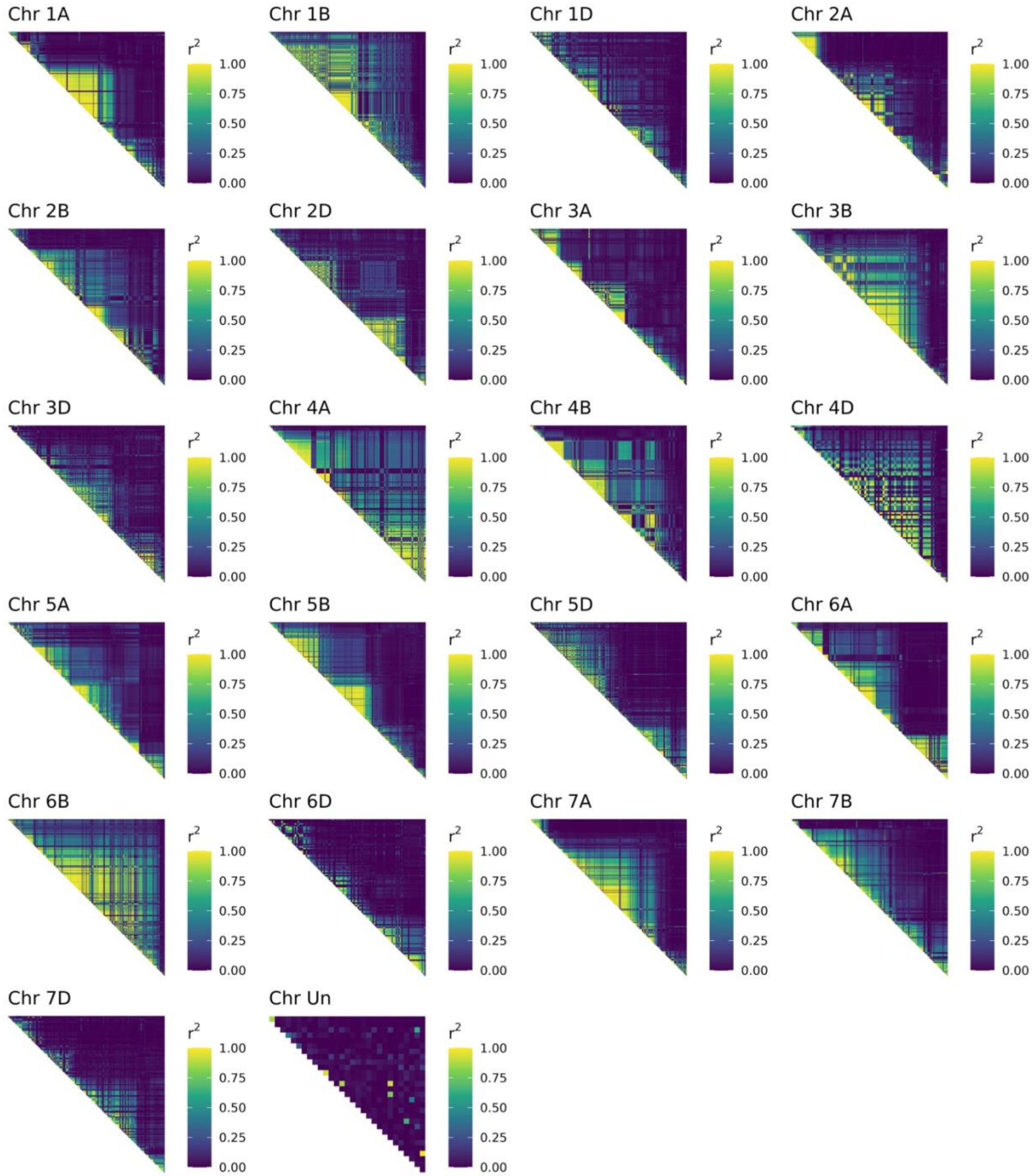

Supplementary Figure S4. Linkage disequilibrium (LD) between SNP markers on each chromosome following marker repositioning. LD was calculated as pairwise correlation (R^2) among 7,032 anchored markers within each chromosome after repositioning.

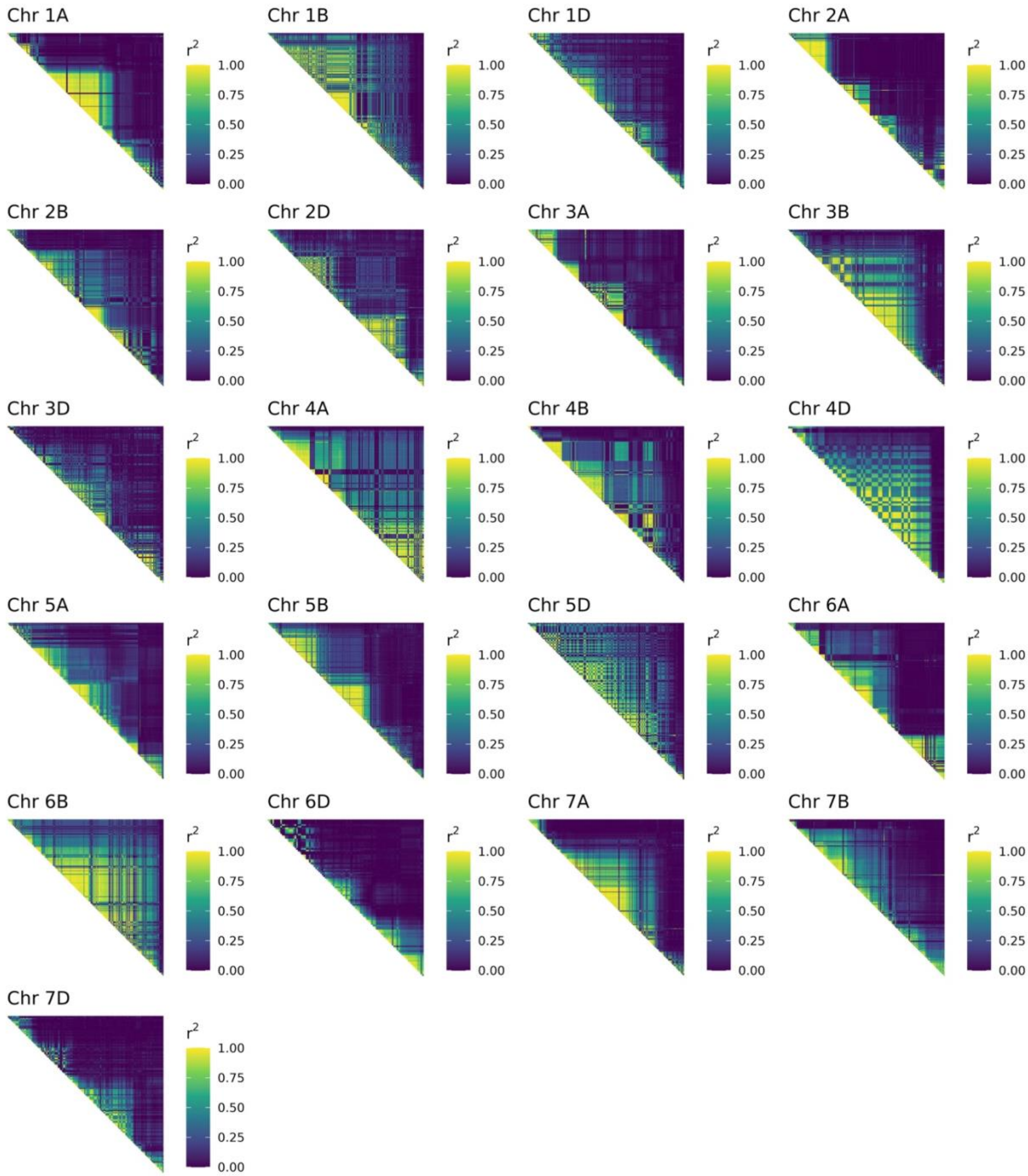

Supplementary Figure S5. Model comparison for GWAS of necrosis development (AUDPC_N_HT74) using alternative fixed-effect covariates. For all GWAS runs, kinship correction was applied using a marker set thinned by LD-based skimming at |*r*| = 1.0. Models were tested with different combinations of fixed covariates: (A) no fixed covariates, (B) family, (C) AABB donor, (D) 10 principal components (10x PC), and (E) family, AABB donor, and 10x PC. For each covariate combination, results are shown as: (i) Manhattan plots, (ii) quantile-quantile (QQ) plots with the corresponding genomic inflation factor (*λ*), and (iii) histograms of GWAS *p*-value distributions.

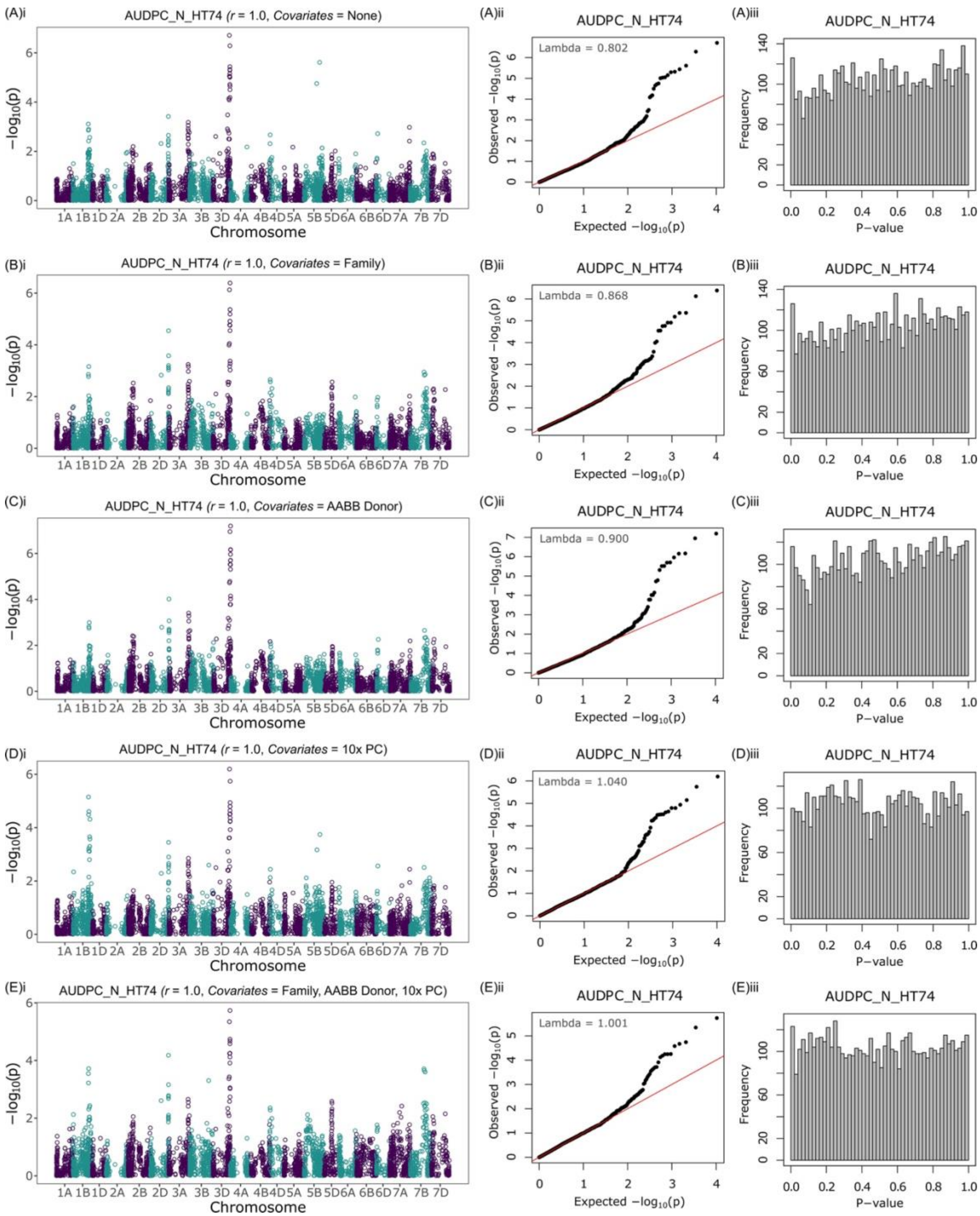

Supplementary Figure S6. Genome-wide linkage disequilibrium (LD) decay in the NAM population. Pairwise LD (r^2) between SNP markers is shown as a function of physical distance (Mb). LD declined to r^2 = 0.2 at approximately 50 Mb; this distance was used to inform interpretation of independent association regions.

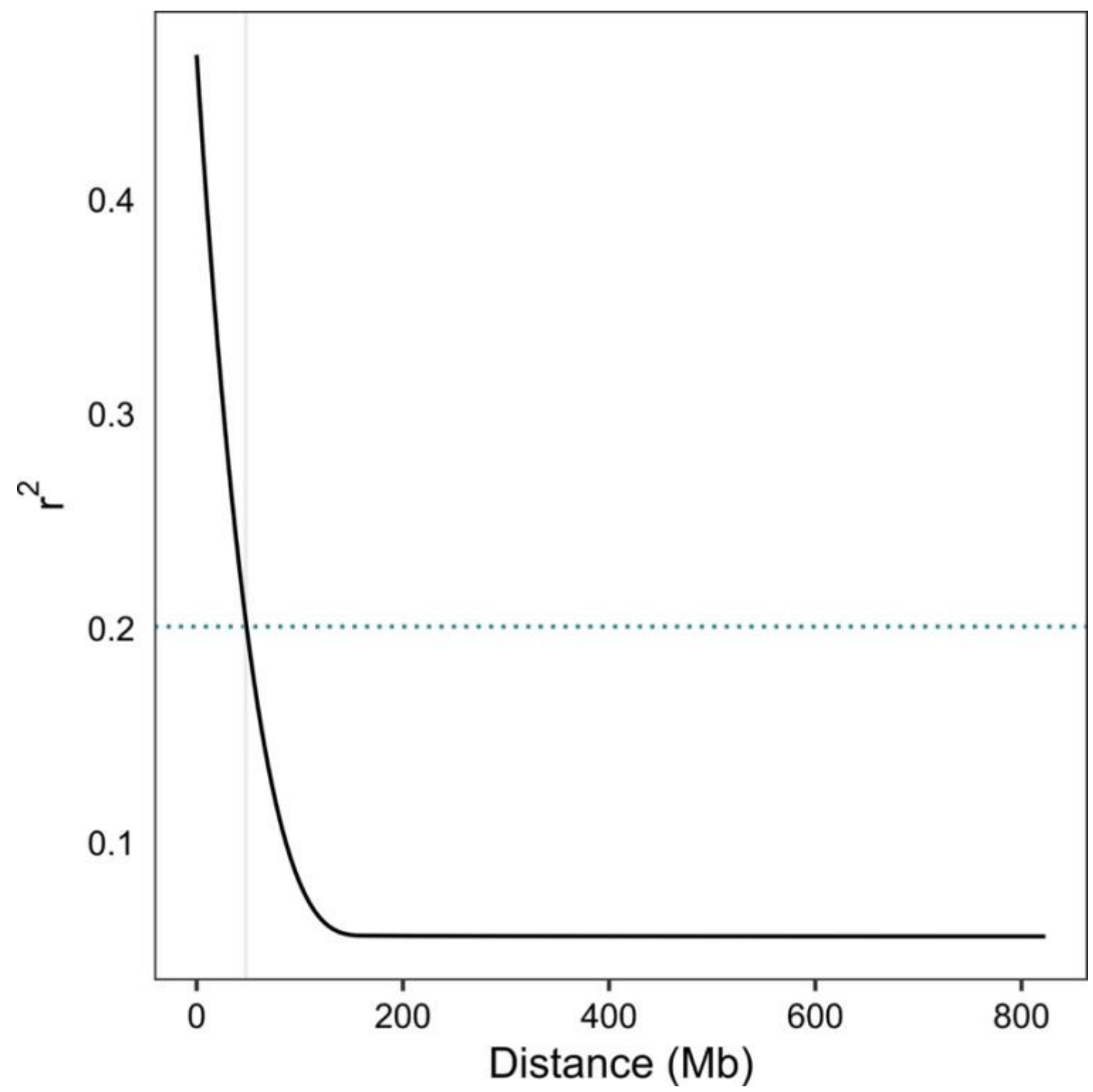

Supplementary Figure S7. Subgenome-specific genetic differentiation among SHW founders. Number of SNPs differentiating each pair of SHW founders (SHW.035, SHW.054, and SHW.075) across the A-, B-, and D-subgenomes. SNPs were classified as differentiating where the two founders carried different allele calls. Bars show the number of differentiating SNPs per subgenome for each pairwise comparison.

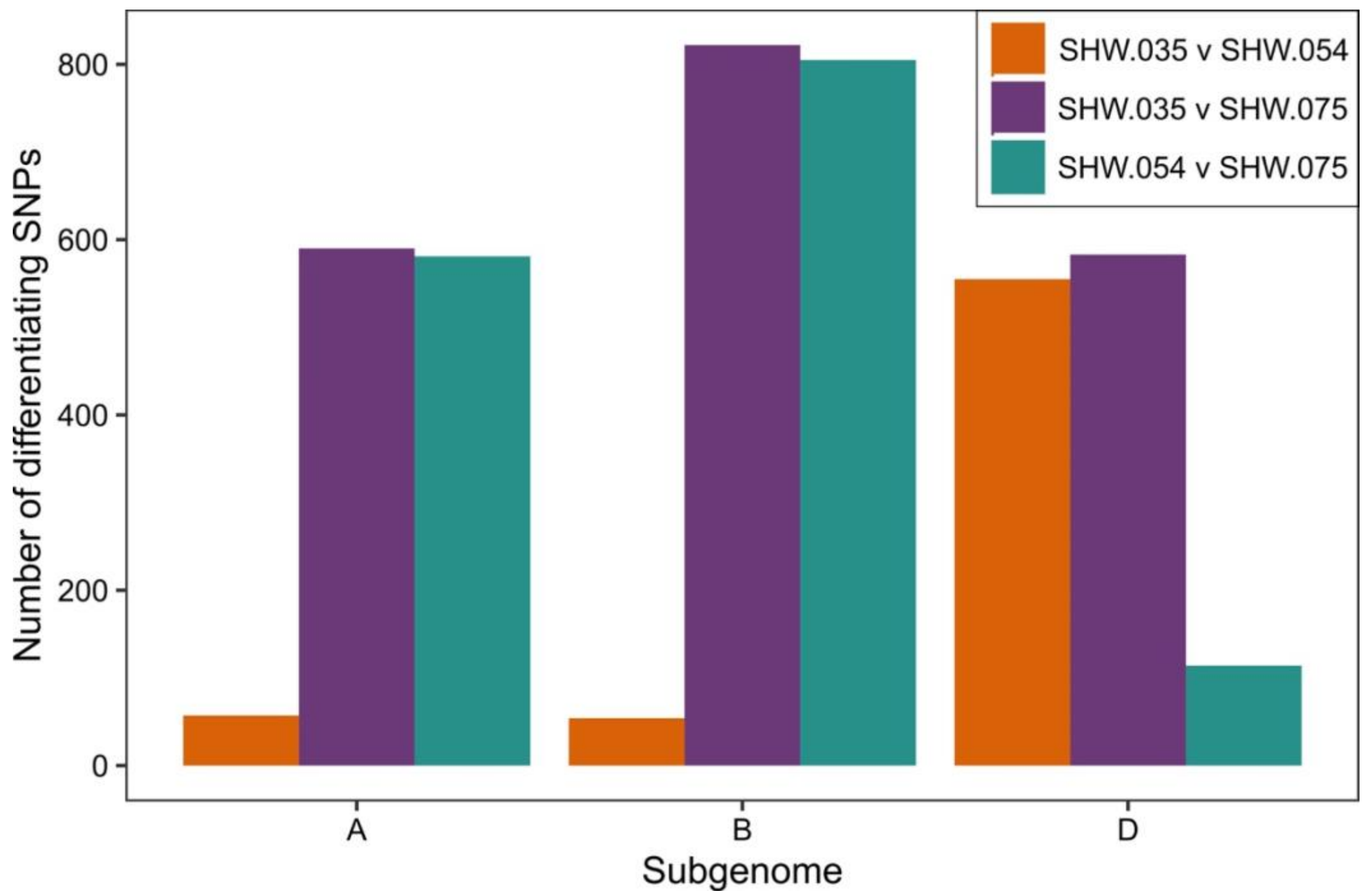

Supplementary Figure S8. Genome-wide association mapping for AUDPC_N across all isolates in the wheat NAM population. Manhattan plots and corresponding quantile-quantile plots show GWAS results for necrosis following inoculation with *Z. tritici* isolates HT-18 (A), HT-44 (B), HT-53 (C), HT-74 (D), and HT-96 (E). The dashed grey horizontal line indicates the genome-wide significance threshold determined by 1,000 permutations at $\alpha = 0.05$ for each isolate, while the dotted yellow line denotes the false discovery rate threshold at $q = 0.05$.

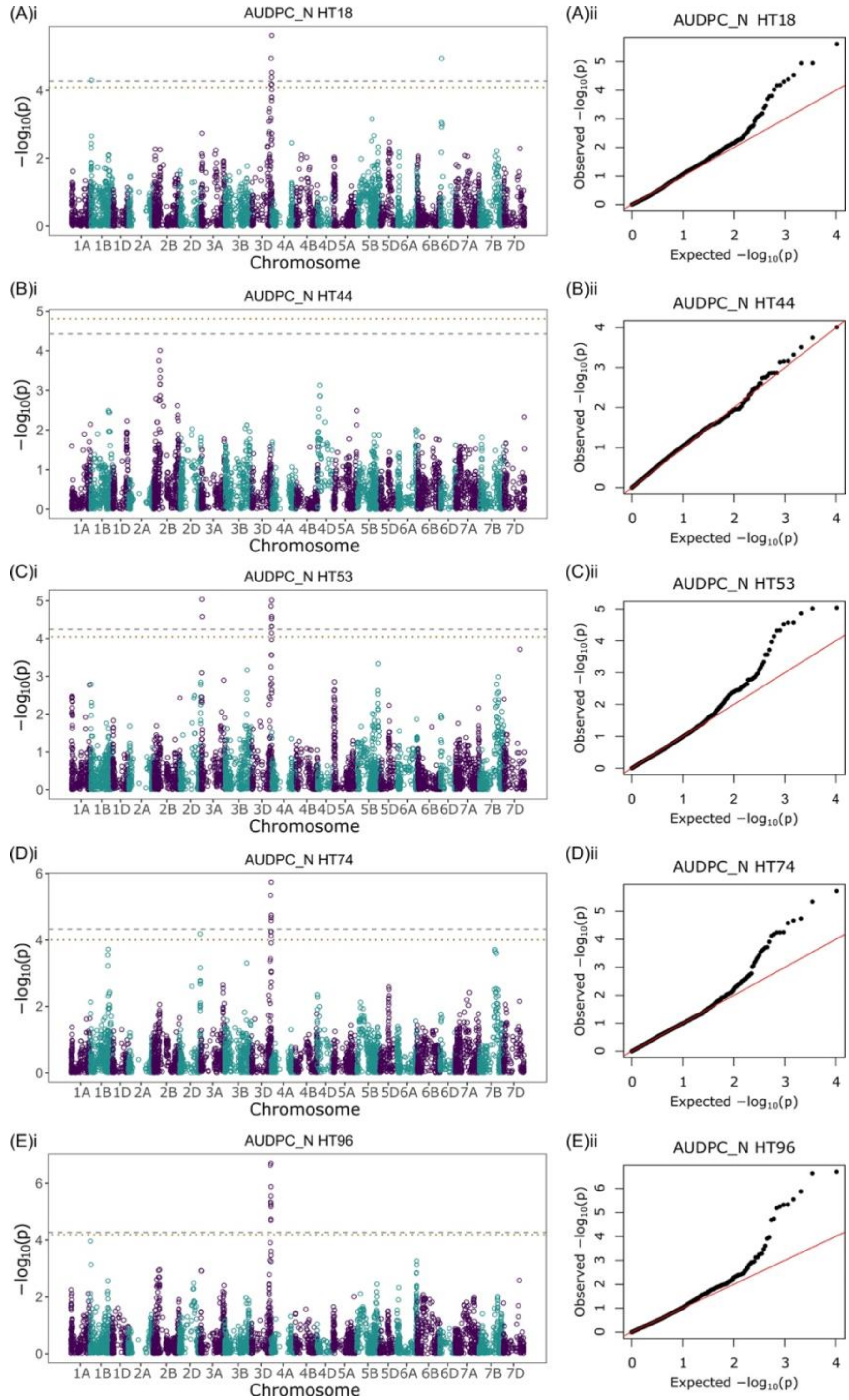

Supplementary Figure S9. Genome-wide association mapping for AUDPC_P across all isolates in the wheat NAM population. Manhattan plots and the corresponding quantile-quantile plots show GWAS results for pycnidia following inoculation with *Z. tritici* isolates HT-18 (A), HT-44 (B), HT-53 (C), HT-74 (D), and HT-96 (E). The dashed grey horizontal line indicates the genome-wide significance threshold determined by 1,000 permutations at $\alpha = 0.05$ for each isolate, while the dotted yellow line denotes the false discovery rate threshold at $q = 0.05$.

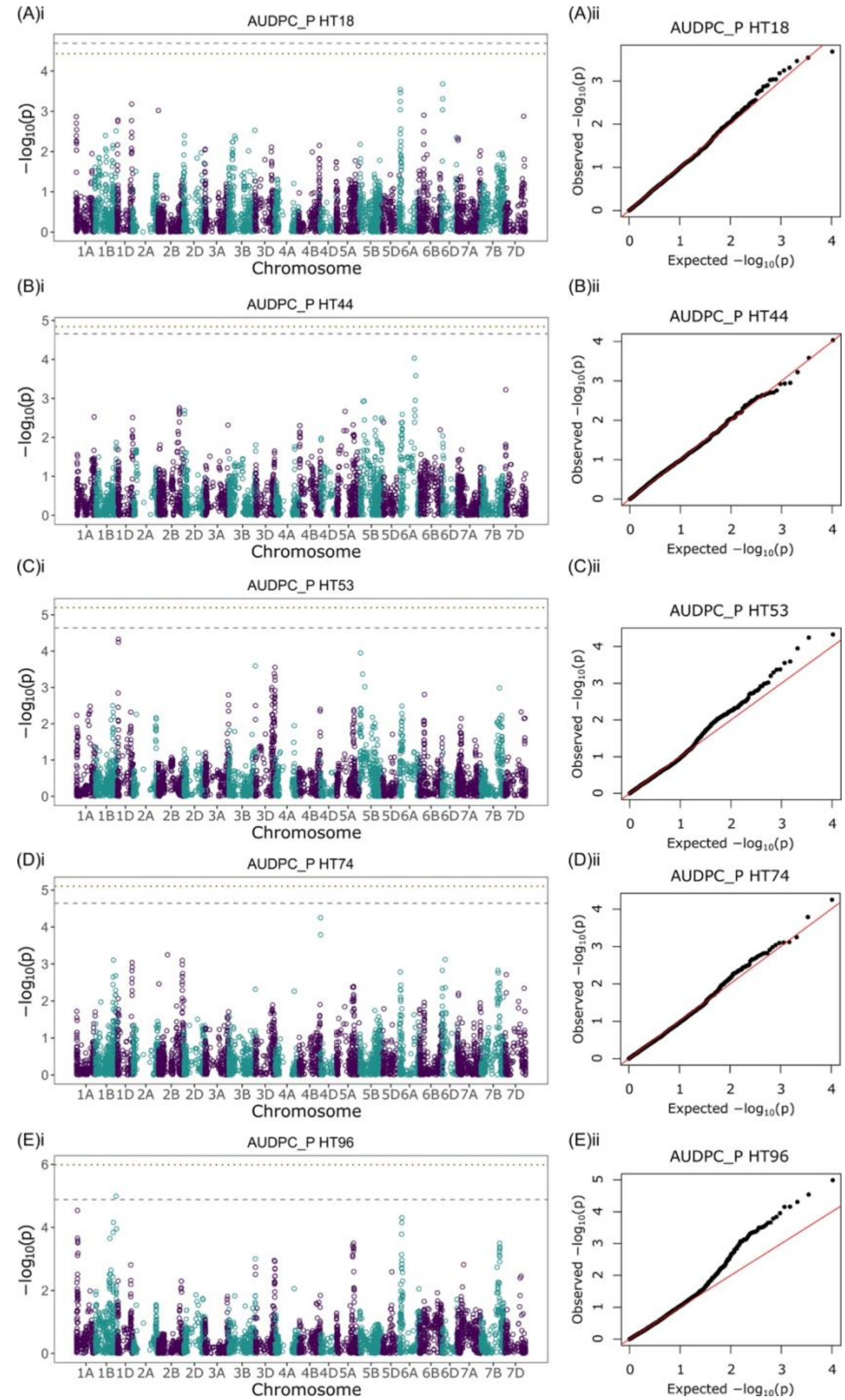

Supplement: Supplementary file 1 [file 122_2026_5423_MOESM2_ESM.pdf]
